# Transgenic Analysis of the *Leishmania* MAP Kinase MPK10 Reveals an Auto-inhibitory Mechanism Crucial for Stage-Regulated Activity and Parasite Viability

**DOI:** 10.1371/journal.ppat.1004347

**Published:** 2014-09-18

**Authors:** Mathieu Cayla, Najma Rachidi, Olivier Leclercq, Dirk Schmidt-Arras, Heidi Rosenqvist, Martin Wiese, Gerald F. Späth

**Affiliations:** 1 Institut Pasteur and Centre National de la Recherche Scientifique URA 2581, Unité de Parasitologie Moléculaire et Signalisation, Paris, France; 2 Strathclyde Institute of Pharmacy and Biomedical Sciences, University of Strathclyde, Glasgow, Scotland; 3 Protein Research Group, Department of Biochemistry and Molecular Biology, University of Southern Denmark, Odense, Denmark; Hebrew University-Hadassah Medical School, Israel

## Abstract

Protozoan pathogens of the genus *Leishmania* have evolved unique signaling mechanisms that can sense changes in the host environment and trigger adaptive stage differentiation essential for host cell infection. The signaling mechanisms underlying parasite development remain largely elusive even though *Leishmania* mitogen-activated protein kinases (MAPKs) have been linked previously to environmentally induced differentiation and virulence. Here, we unravel highly unusual regulatory mechanisms for *Leishmania* MAP kinase 10 (MPK10). Using a transgenic approach, we demonstrate that MPK10 is stage-specifically regulated, as its kinase activity increases during the promastigote to amastigote conversion. However, unlike canonical MAPKs that are activated by dual phosphorylation of the regulatory TxY motif in the activation loop, MPK10 activation is independent from the phosphorylation of the tyrosine residue, which is largely constitutive. Removal of the last 46 amino acids resulted in significantly enhanced MPK10 activity both for the recombinant and transgenic protein, revealing that MPK10 is regulated by an auto-inhibitory mechanism. Over-expression of this hyperactive mutant in transgenic parasites led to a dominant negative effect causing massive cell death during amastigote differentiation, demonstrating the essential nature of MPK10 auto-inhibition for parasite viability. Moreover, phosphoproteomics analyses identified a novel regulatory phospho-serine residue in the C-terminal auto-inhibitory domain at position 395 that could be implicated in kinase regulation. Finally, we uncovered a feedback loop that limits MPK10 activity through dephosphorylation of the tyrosine residue of the TxY motif. Together our data reveal novel aspects of protein kinase regulation in *Leishmania*, and propose MPK10 as a potential signal sensor of the mammalian host environment, whose intrinsic pre-activated conformation is regulated by auto-inhibition.

## Author Summary

Leishmaniasis is an important human disease caused by *Leishmania* parasites. A crucial aspect of *Leishmania* infectivity is its capacity to sense different environments and adapt for survival inside insect vector and vertebrate host by stage differentiation. This process is triggered by environmental changes encountered in these organisms, including temperature and pH shifts, which usually are sensed and transduced by signaling cascades including protein kinases and their substrates. In this study, we analyzed the regulation of the *Leishmania* mitogen-activated protein kinase MPK10 using protein purified from transgenic parasites and combining site-directed mutagenesis and activity tests. We demonstrate that this kinase is activated during parasite differentiation and regulated by an atypical mechanism involving auto-inhibition, which is essential for parasite viability.

## Introduction

Leishmaniasis is an infectious disease characterized by a variety of pathologies, affecting more than 12 million people worldwide and ranging from self-healing cutaneous lesions to fatal visceral infection [1]. This disease is caused by pathogenic protozoa of the genus *Leishmania*, which show two major life cycle stages depending on the host. The extracellular promastigote stage develops inside the midgut of sandflies and is transmitted during blood feeding to a vertebrate host where they are ingested by phagocytic cells, notably macrophages. Inside the host cell phagolysosome, promastigotes develop into proliferating intracellular amastigotes. These developmental transitions are triggered by environmental changes, mainly pH (7.4 to 5.5) and temperature (26°C to 37°C), encountered in insect and vertebrate hosts, respectively, and can be mimicked in vitro [2]–[4]. Interfering with amastigote stage development and proliferation by altering the parasite's ability to sense its environment could be a very efficient way to eliminate intracellular *Leishmania* and thus signaling proteins involved in extra- or intracellular signal transduction are interesting drug target candidates.

In eukaryotes, environmental signals are generally sensed and transduced by signaling cascades involving receptors and downstream-regulated protein kinases. The MAPK signaling pathway is a good example of such a phosphorylation cascade [5] as it is composed of mitogen-activated protein kinase kinase kinases (M3Ks), which activate mitogen-activated protein kinase kinases (M2Ks), which in turn activate mitogen-activated protein kinases (MAPKs) by dual phosphorylation on the highly conserved TxY motif present within the MAPK activation loop [6], [7]. MAPKs regulate various important cellular functions, such as cell cycle progression and differentiation, through phosphorylation of a large number of substrates, including transcription factors and MAPK-activated protein kinases, thus modifying gene expression and post-translational regulation, respectively [8]–[14]. While the core cascade M3K-M2K-MAPK is conserved in *Leishmania*, the absence of classical transcription factors and the largely constitutive gene expression suggest that the response to environmental signals occurs mainly post-translationally through the regulation of the level of protein phosphorylation, rather than through modulation of gene expression [15].

As in higher eukaryotes, the *Leishmania* MAPK pathway comprises two distinct kinase families, the STE family, which includes five putative M2K and M2K-like and 20 putative M3K members, and the CMGC family, including 17 putative MAPK and MAPK-like members [16]. The comparison between the *Leishmania major* and the human kinomes revealed an evolutionary expansion relative to genome size of these two kinase families in the parasite. The STE and CMGC families represent 19% and 25% in the *Leishmania* kinome, compared to 9% and 12% in the human kinome, respectively [17]. These expansions indicate that the MAPK pathway could be of particular importance to parasite development and survival, a possibility that is supported by recent investigations showing that *Leishmania* MAP kinases are required for flagellar development, intracellular survival and viability [16],[18]–[23]. Thus, the study of *Leishmania* MAPKs could hold the key to the understanding of the mechanisms that allow the adaptation of *Leishmania* to environmental changes required for extra- and intracellular parasite survival during host infection.

The activity of the three *Leishmania* MAPKs MPK4, MPK7 and MPK10 have been shown to be induced in a stage-specific manner in axenic amastigotes, which occurred concomitant to an increase of their phosphorylation as expected for this class of kinases [24]–[26]. The importance of MPK4 and MPK7 for cell survival and infectivity, respectively, has been established [21], [26], [27], but neither the mode of regulation nor the functions of MPK10 have been studied. MPK10 is conserved in all *Leishmania* species (Figure S1A) with a percentage of identity above 90%. This percentage is even higher if we compare the sequence of *L. donovani* MPK10 with that of *L. major*, *L. mexicana* and *L. infantum* (99%, 98% and 100%, respectively, Figure S1B). The close relationship between this kinase and human ERK2 or p38 kinase suggests a potential role in cell differentiation or in response to stress [28], [29]. *Leishmania* MPK10 is a highly conserved member of the eukaryotic MAPK family. However, MPK10 does not resemble a classical eukaryotic MAPK with respect to two structural features. First, the alignment of the protein sequence with its mammalian orthologs revealed the presence of a long carboxy-terminal extension [16], [24]. This is a common feature for *Leishmania* MAPKs (observed in 15 out of 17 putative *Leishmania* MAPKs), whereas only five mammalian MAPKs have such an extension. They are usually regulatory domains implicated in the control of the kinase activity, localization or auto-inhibition [30]–[34]. Second, a structural analysis of MPK10 published recently by Horjales *et al.* provided evidence that recombinant MPK10 adopts an activated conformation, despite the absence of TxY phosphorylation [29]. Following phosphorylation of this motif by upstream M2Ks, mammalian MAPKs are activated by the switch of a conserved DFG motif from an inactive (DFG-out) to an active (DFG-in) conformation. This change leads to the alignment of two structural motifs comprising non-consecutive hydrophobic residues that are referred to as the regulatory and catalytic spines [35], [36]. In contrast to mammalian MAPKs, the *Leishmania* DFG motif of MPK10 is replaced by DFN, causing the R and C spines to be aligned thus stabilizing an apparent active conformation in the absence of TxY phosphorylation [29]. These findings suggest that MPK10 has a strikingly different mode of regulation compared to mammalian MAPKs, which could be particularly adapted to adjust quickly to environmental changes thus acting as a signaling switch.

Here, by utilizing a transgenic approach we unraveled an unusual mechanism of MPK10 regulation that tightly controls kinase activity. Stage-specific increase in MPK10 activity during the pro- to axenic amastigote conversion did not follow the largely constitutive phosphorylation status of the regulatory tyrosine residue in the kinase activation loop, suggesting additional and non-classical mechanisms of MPK10 regulation. Combining limited tryptic digestion with mutagenesis analysis and phosphoproteomics investigation, we uncovered an essential role of the C-terminal domain of MPK10 in limiting stage-specific kinase activity, and identified a novel regulatory phospho-serine residue that is required for axenic amastigote viability. Together our data propose MPK10 as a potential signal sensor of the mammalian host environment whose intrinsic pre-activated conformation is regulated by auto-inhibition. These results shed important new light on *Leishmania*-specific signaling mechanisms, and significantly advance our understanding on parasite-specific protein kinase biology.

## Results

### Recombinant MPK10 shows no significant substrate-specific kinase activity

**Figure 1:**
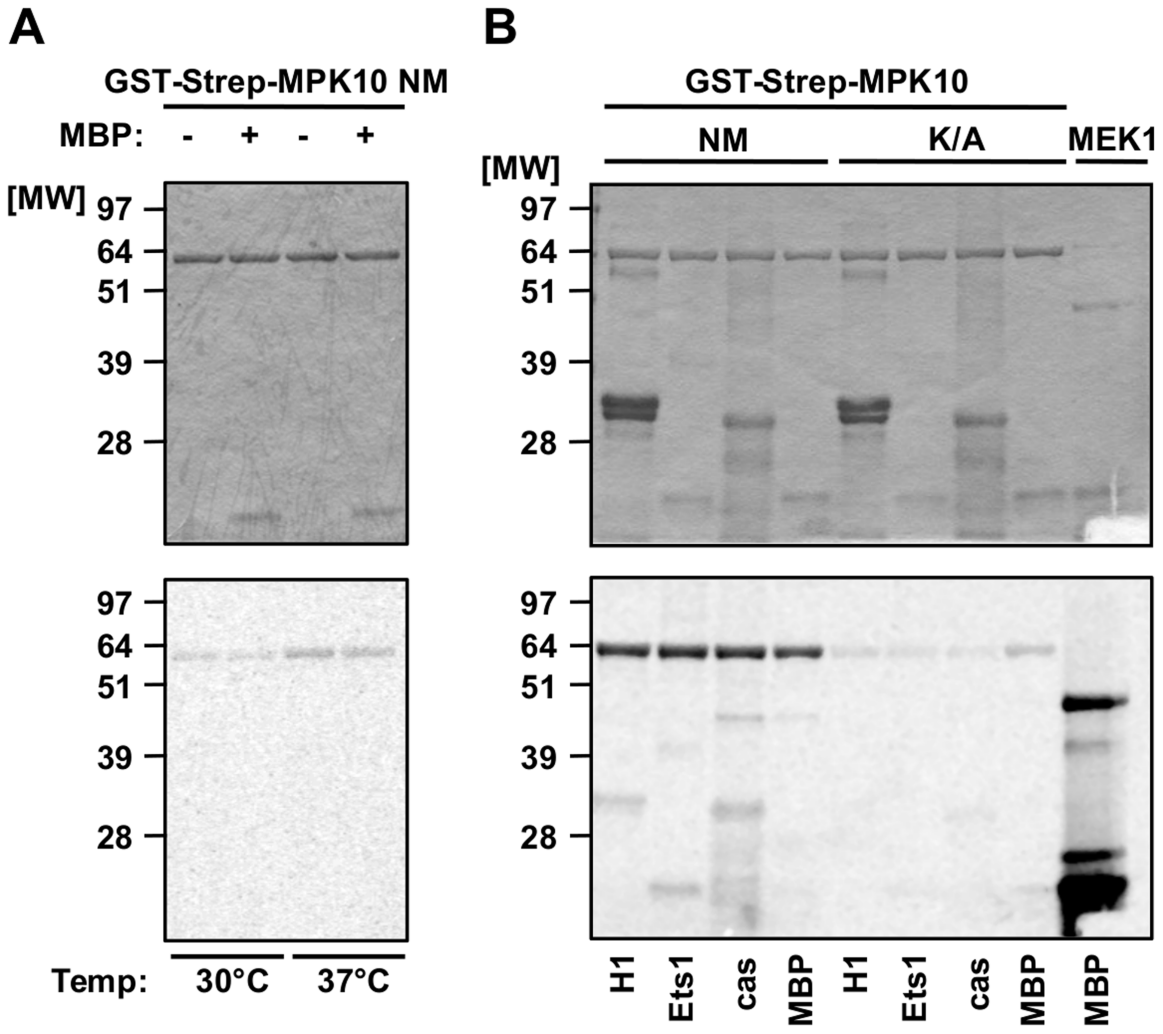
Recombinant MPK10 shows no significant substrate-specific kinase activity. *In vitro* kinase assays using non-mutated GST-Strep-MPK10 (NM) and the corresponding inactive mutant GST-Strep-MPK10-K51A (K/A). Analysis of the reaction samples was performed by SDS-PAGE, gels were stained with Coomassie (upper panels) and phosphotransfer was visualized by auto-radiography (lower panels). All gels are representative of three independent experiments. (**A**) Purified GST-Strep-MPK10 NM was incubated with (+) or without (−) the canonical MAPK substrate MBP for 30 min at pH 7.5 and 30°C or 37°C. (**B**) GST-Strep-MPK10 NM and -K/A were incubated with different canonical substrates, including 12 µg of histone H1, 9 µg of Ets1, 36 µg of casein, and 25 µg of MBP. Recombinant human MEK1 was used as positive control with MBP as substrate. Kinase assays were performed for 30 min at pH 7.5 and 37°C.

To investigate the biochemical properties of MPK10, we generated non-mutated recombinant MPK10 (NM) and the corresponding MPK10-K51A enzymatically dead mutant, both tagged with GST-Strep. We used the bacterially expressed and purified proteins in an *in vitro* kinase assay at 30°C or 37°C, monitoring the transfer of radiolabeled phosphate from γ-^33^P-ATP to MPK10 (auto-phosphorylation) or to the canonical MAPK substrate myelin basic protein (MBP) [37]–[39]. The kinase reactions were subjected to SDS-PAGE, proteins were visualized by Coomassie staining for normalization (Figure 1A, upper panel), and phosphotransferase activity was revealed by auto-radiography (Figure 1A, lower panel). We observed a weak auto-phosphorylation signal at 30°C that was slightly enhanced when the kinase reaction was performed at 37°C (Figure 1A, lower panel). We did not observe any significant activity towards MBP at both temperatures. Likewise, performing kinase assays at 37°C and pH 7.5 with other substrates, including casein, histone H1 or Ets1, revealed only a very weak substrate-specific activity, with casein giving rise to the strongest signal, which still was faint compared to the levels of auto-phosphorylation (Figure 1B, lower panel). By contrast, recombinant human MEK1 (a M2K), used as a positive control, revealed strong substrate-specific activity towards MBP. No substrate phosphorylation or auto-phosphorylation could be detected with the kinase dead MPK10-K51A control, suggesting that the signals observed were specific for MPK10 and not due to a co-purified bacterial kinase. Based on these results, casein appears to be the most suitable substrate for recombinant MPK10.

We next varied the pH of the kinase assay in an attempt to improve MPK10 activity. Kinase assays performed with casein and MPK10 or MPK10-K51A at pH 5.5, 6.5, 7.5 and 8.5 revealed different pH optima for auto- and substrate-specific phosphorylation at pH 6.5 and 7.5, respectively (Figure S2, lower panel). In conclusion, recombinant MPK10 shows some minor auto-phophorylation activity, but largely fails to phosphorylate the canonical MAPK substrate MBP [37]–[39], which may either depend on activation through an upstream M2K, parasite-specific kinase substrate interactions, or auto-inhibition.

### Deletion of a C-terminal domain increases MPK10 kinase activity

**Figure 2:**
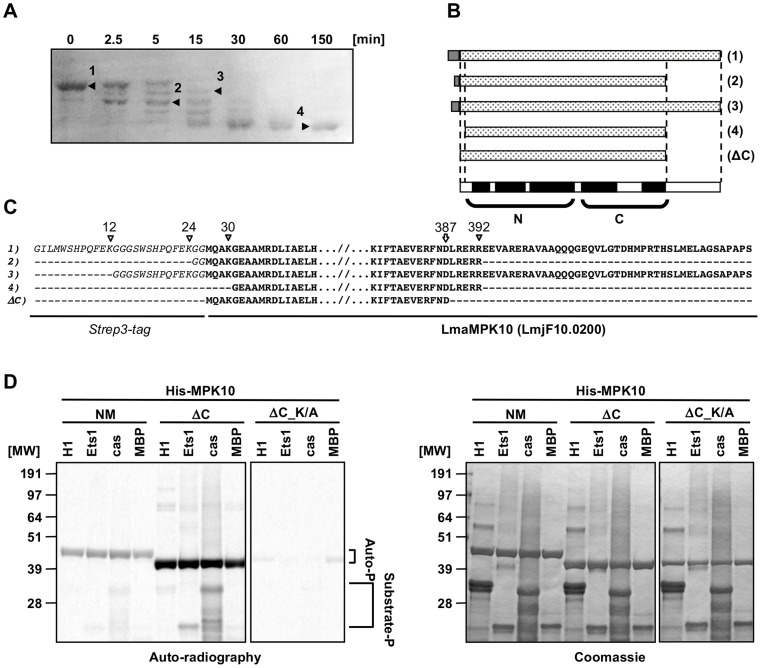
Deletion of the C-terminal domain increases auto-phosphorylation activity. (**A**) Partial tryptic digestion of recombinant MPK10. 50 µg of Strep3-MPK10 were digested with 0.25 µg trypsin at RT. Aliquots were taken at the indicated time points and the reaction was stopped either by adding Laemmli buffer (for N-terminal sequencing) or by lowering the pH to 5.0 and subsequent freezing (for mass determination by SELDI-TOF). For N-terminal sequencing, samples were separated by SDS-PAGE, transferred on PVDF membrane and stained by amidoblack. N-terminal sequencing was performed at the protein analysis platform at the Institut Pasteur. For mass determination, samples were immobilized on a H4 ProteinChip Array (C16 reversed phase surface) and peptide masses identified by SELDI-TOF. Results of the N-terminal sequencing are represented by the cartoon in (**B**), and the sequences are indicated in (**C**). Italic characters represent the Strep3-tag and bold characters represent the sequence of *Leishmania major* MPK10. White and grey arrowheads indicate respectively lysine or arginine residues recognized by trypsine, including K12, K24, K30 and R392. The white arrow at the position D387 indicates the position of the last cleaved residue resulting in the generation of the form lacking the last 46 amino acids of MPK10. (**D**) *In vitro* kinase assay using recombinant His-MPK10 (NM) and the truncated kinase mutants His-MPK10-ΔC (ΔC), and His-MPK10-ΔC_K51A (ΔC_K/A). Results are representative of three independent experiments. Purified proteins were incubated with four different substrates, including 12 µg of histone H1, 9 µg of Ets1, 36 µg of casein, and 25 µg of MBP. Recombinant human MEK1 was used as positive control with MBP as substrate. Kinase assays were performed at the same time for 30 min at pH 7.5 and 37°C and reaction samples were separated by SDS-PAGE, gels were stained by Coomassie (right), and signals were revealed by auto-radiography with the same exposure time between the different gels (left). The brackets in (D) indicate auto-phosphorylation (Auto-P) and substrate phosphorylation (Substrate-P) signals.

We performed a limited tryptic digestion of purified recombinant GST-Strep-tagged MPK10 to investigate the presence of potential auto-inhibitory accessory domains by delineating the structured, protease-resistant kinase core. We treated recombinant MPK10 with 0.25 µg of trypsin for 150 minutes, analyzing samples after 2.5, 5, 15, 30, 60 and 150 minutes by SDS-PAGE and Coomassie staining. MPK10 was sensitive to partial digestion as we observed the appearance of several bands within the first 15 minutes corresponding to different tryptic MPK10 products (Figure 2A). After 30 min of treatment, only one band remained, revealing the core of the kinase. N-terminal sequencing and SELDI-TOF analysis of four of the digestion products (as marked by arrowheads in Figure 2A and represented by the cartoon shown in Figure 2B) revealed that both ends of the tagged protein were cleaved by trypsin at lysines 12, 24, and 30, arginine 392. We also identified a digestion product that resulted from cleavage at aspartate 387 and thus lacked the last 46 amino acids of MPK10 (Figure 2C). This product is likely due to a miscleavage or cleavage by a contaminating bacterial protease.

To investigate the activity of the MPK10 kinase core alone and to test whether deletion of the C-terminal region (46 aa) increases its activity, we generated and purified recombinant non-mutated His_6_-MPK10 (NM) or mutated His_6_-MPK10 deleted for the last 46 amino acids (ΔC) and monitored their activity towards canonical substrates by performing *in vitro* kinase assays in the presence of γ-^33^P-ATP. We changed from the GST-Strep to His_6_ tag to show that the lack of MPK10 substrate phosphorylation activity is independent from this modification. The auto-radiogram shown in Figure 2D (left panel) confirms weak auto- and substrate phosphorylation activity of His_6_-MPK10 NM similar to GST-STREP-MPK10 NM ruling out tag-specific interference with kinase activity. In contrast to His_6_-MPK10 NM, His_6_-MPK10-ΔC presented a stronger auto-phosphorylation activity and enhanced phosphorylation of Ets1 and casein. These differences reflect a true increase in phosphotransferase activity as judged by Coomassie staining, which showed equal loading of both recombinant proteins and substrates (Figure 2D, right panel). The signals were specific for His_6_-MPK10-ΔC and not caused by a co-purified kinase, as we did not detect any signal with the His_6_-MPK10-ΔC_K51A control. Yet again, the auto-phosphorylation signals of NM or truncated MPK10 were significantly stronger than those for substrate phosphorylation. Moreover, no ^33^P incorporation could be detected for MBP. Thus, deleting the C-terminal tail substantially increases the ability of MPK10 to phosphorylate itself, suggesting a potential role of this domain in negative regulation. The C-terminal domain of *L. donovani* MPK10 is conserved in *Leishmania* species (84 to 100%, Figure S3B) but not in *Trypanosoma* species (38 to 53%, Figure S3B), except for the conserved motif (DHMxRTxSxME), of unknown function (underlined, Figure S3A). This motif is only conserved in trypanosomatid MPK10 as extending the analysis of the C-terminal extensions to the 14 *Leishmania* and 2 human MAPKs (ERK5 and ERK8) as well as *T. brucei* TbECK1 [32] by pattern recognition analysis (Pratt version 2.1) and multiple sequence alignment (ClustalW) did not reveal this motif or any other conserved motifs or patterns (data not shown). As our study reveals important limitations of the bacterially expressed protein with regard to kinase activity, we analyzed in the following *in situ* activated MPK10 isolated from transgenic *L. donovani* parasites.

### MPK10 activity is substantially increased during axenic amastigote differentiation

**Figure 3:**
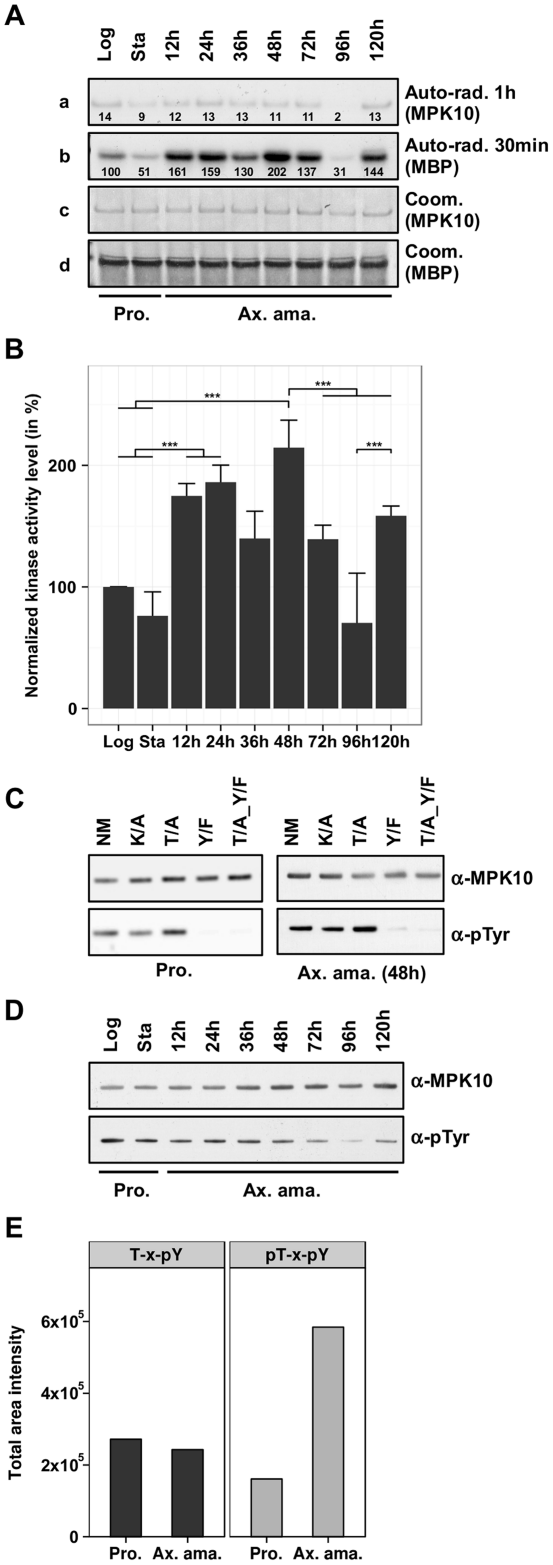
MPK10 activity is enhanced during axenic amastigote differentiation. (**A**) *In vitro* kinase assay. GFP-MPK10 NM fusion protein was purified using anti-GFP antibody from logarithmic (Log) and stationary (Sta) growth phase promastigotes (Pro) and after 12 h, 24 h, 36 h, 48 h, 72 h, 96 h and 120 h of axenic amastigote (Ax. ama.) differentiation. Kinase assays were performed for 30 min at 37°C with 25 µg of MBP as substrate. Reaction samples were separated by SDS-PAGE, gels were stained with Coomassie (Coom.), and signals were revealed by auto-radiography (Auto-rad.) using the indicated exposure time. The numbers represent the level of radioactive counts as determined in a scintillation counter. The results were expressed relative to GFP-MPK10 NM from promastigotes of logarithmic culture set to 100%. (**B**) Signal quantification. The histogram plot represents the level of radioactive counts as determined in a scintillation counter. The results were expressed relative to GFP-MPK10 NM from promastigotes of logarithmic culture set to 100%. Histograms represent mean values and the error bars correspond to the standard deviation of three independent experiments. Statistical significance was calculated by t-test or Mann-Whitney Rank Sum test. (**C**) Assessment of tyrosine phosphorylation. Non-mutated GFP-MPK10 (NM), and mutated GFP-MPK10-K51A (K/A), -Y192F (Y/F) and -T190A_Y192F (T/A_Y/F) were purified from promastigote (Pro.) and axenic amastigote (Ax. ama.) after 48 h of differentiation, and analyzed by western blotting using anti-phospho-tyrosine (α-pTyr) and anti-MPK10 (α-MPK10). (**D**) Analysis of tyrosine phosphorylation during axenic amastigote differentiation. Samples used in (A) were analyzed by western blotting using mouse anti-phospho-tyrosine (α-pTyr) and rabbit anti-MPK10 (α-MPK10) antibodies. (**E**) SRM analysis. Digested proteins from whole cell lysates of *L. mexicana* promastigotes (Pro) and axenic amastigotes (Ax. Ama), were subjected to TiO_2_ enrichment, eluates were desalted on C8 STAGE-tips and vacuum dried. Prior to LC-MS/MS analysis, the eluates were reconstituted in 0.1% formic acid. The acquired data were imported into the Pinpoint method file for analysis. T-H-pY, peptides represent single phosphorylation of Y192; pT-H-pY, peptides represent dual phosphorylation of residues T190 and Y192. This value of T-H-pY represents the sum of the values obtained with two different peptides (THYVTHR and EDTADANKTHYVTHR). All blots and auto-radiograms are representative of three independent experiments.

To gain insight into MPK10 regulation and activity in a physiologically relevant context, we generated transgenic parasites expressing a GFP-MPK10 fusion protein from the episomal vector pXG-GFP2+ (kindly provided by S. Beverley). We first performed a time course experiment to investigate MPK10 activity in promastigotes and during axenic amastigote differentiation. GFP-MPK10 was purified using a monoclonal anti-GFP antibody from *L. donovani* promastigotes harvested from logarithmic (log) or stationary (stat) phase cultures, and from cultures at different time points between 12 h to 120 h after induction of axenic amastigote differentiation by pH and temperature shift. Immuno-purified proteins were incubated for 30 min at 37°C in the presence of radiolabeled ATP and MBP, and the kinase reaction was subjected to SDS-PAGE. The phosphotransferase activity was determined by auto-radiography (Figure 3A, a and b), and MPK10 and MBP were visualized by Coomassie staining for normalization (Figure 3A, c and d). After exposure, the bands corresponding to the signals of phosphorylated MPK10 or MBP were recovered from the dried gel and ^32^P incorporation was measured using a scintillation counter. The results were expressed relative to GFP-MPK10 NM from promastigotes of logarithmic culture set to 100% (the relative counts are represented by the numbers in Figure 3A a and b). As opposed to recombinant MPK10 we observed that MBP phosphorylation was higher than MPK10 auto-phosphorylation when using protein purified from parasite extracts. This finding suggests that the purification of GFP-MPK10 from transgenic parasites allows the assessment of biologically relevant kinase activity. MPK10 purified from parasites undergoing axenic amastigote differentiation showed a higher level of MBP phosphorylation compared to promastigotes, especially during the first 48 h following temperature and pH shift (202% versus 100% respectively, Figure 3A, b). Afterwards, the level of activity decreases to reach its lowest point at 96 h (31%), and increases again at 120 h (144%). These data suggest that MPK10 activity is stage-specifically regulated. The signals observed are specific to MPK10 and not due to a co-purified kinase as no signals were observed with the K51A mutant (data not shown).

The mean values with standard deviation of three independent experiments are represented by the histogram plot shown in Figure 3B. As judged by statistical analysis (see Figure S4), significant differences in MPK10 kinase activity are observed (i) between promastigotes and parasites at 48 h of axenic amastigote differentiation confirming its stage-specific regulation, (ii) between parasites at 48 h and 72 h or 96 h, demonstrating a transient reduction in MPK10 activity during later stages of axenic differentiation, and (iii) between parasites at 96 h and 144 h providing evidence for a second peak in MPK10 activity in axenic amastigotes. Conversely, no significant difference in MPK10 activity was observed between promastigotes from logarithmic or stationary phase culture.

Phosphorylation of both the threonine and the tyrosine residues of the TxY motif have been shown to be required for eukaryotic MAPK activation and dual phosphorylation is correlated with kinase enzymatic activity. As an alternative read out for MPK10 activity we therefore assessed tyrosine phosphorylation using an anti-phosphotyrosine antibody. First, we tested whether phosphorylated Tyr192 was the only residue recognized by the antibody in MPK10. We compared the signals obtained with GFP-MPK10 NM, GFP-MPK10-K51A and the corresponding TxY motif mutants T190A, Y192F and T190A_Y192F purified from respective transgenic promastigotes harvested in logarithmic growth phase (low level of MPK10 activity, Figure 3B), or cells during axenic amastigotes differentiation harvested at 48 h after induction of differentiation (high level of MPK10 activity, Figure 3Ab and B). The western blot presented in Figure 3C shows that GFP-MPK10 NM, GFP-MPK10-K51A and GFP-MPK10-T190A are recognized by the anti-phosphotyrosine antibody as documented by the detection of a strong signal at 75 kDa (Figure 3C). The absence of this signal in GFP-MPK10-Y192F and GFP-MPK10-T190A_Y192F demonstrates that pY192 is the only residue recognized by this antibody in GFP-MPK10. Moreover, Y192 phosphorylation does not require T190 phosphorylation as Y192 is phosphorylated in the GFP-MPK10-T190A single mutant. Remarkably, we did not observe any difference in the level of tyrosine phosphorylation between MPK10 purified from log promastigotes or from 48 h axenic amastigotes despite their difference in activity, suggesting that the phosphorylation state of Y192 is dissociated from the regulation of kinase activity. In contrast, no tyrosine phosphorylation was observed for recombinant MPK10 NM, which further supports the observed inactive state of the bacterially purified kinase (Supplementary Figure S5).

We next studied dissociation of MPK10 activity and Y192 phosphorylation state in a more detailed time course experiment by western blot analysis of pY192 levels in promastigotes (log and stat) and during axenic amastigote differentiation (Figure 3D). Again, no difference was observed in the phosphorylation state of Y192 between MPK10 purified from log promastigotes, stat promastigotes or axenic amastigotes during the first 48 h of differentiation, despite their significant differences in activity. After 72 h we observed a decrease in tyrosine phosphorylation, which this time correlated with decreased MPK10 activity.

These findings were supported by an independent experiment performed with *Leishmania mexicana* using a targeted quantitative phosphoproteomic analysis termed Selected Reaction Monitoring (SRM, [40]), from which results concerning MPK10 were extracted and presented in Figure 3E. In this analysis the phosphorylation states of the two regulatory phosphorylation sites T190 and Y192 were quantified in *L. mexicana* late log phase promastigotes and axenic amastigotes at 72 h of differentiation. Peptides containing T190-H-pY192 (unphosphorylated T190 and phosphorylated Y192) were found at similar levels in promastigotes and axenic amastigotes, whereas peptides containing pT190-H-pY192 (pT190 and pY192 dual phosphorylation) showed significantly increased abundance in the axenic amastigote fraction. Peptides containing a single pT190 phosphorylation were not detected. These results show that phosphorylation of T190 and Y192 occurs independently: while pY192 is identified both in promastigotes and in axenic amastigotes, pT190 is mostly identified in amastigotes and thus this phosphorylation event may be the rate limiting step for MPK10 activation. Altogether, these findings strongly suggest that the phosphorylation of the Y192 residue is largely constitutive and occurs independently from MPK10 activation, which is in contrast to MAPK regulation in most other eukaryotes. This characteristic of MPK10 is likely a conserved feature among *Leishmania* species as we found similar results with independent experiments performed with *L. donovani* and *L. mexicana*.

### Both threonine 190 and tyrosine 192 are required for MPK10 activity

**Figure 4:**
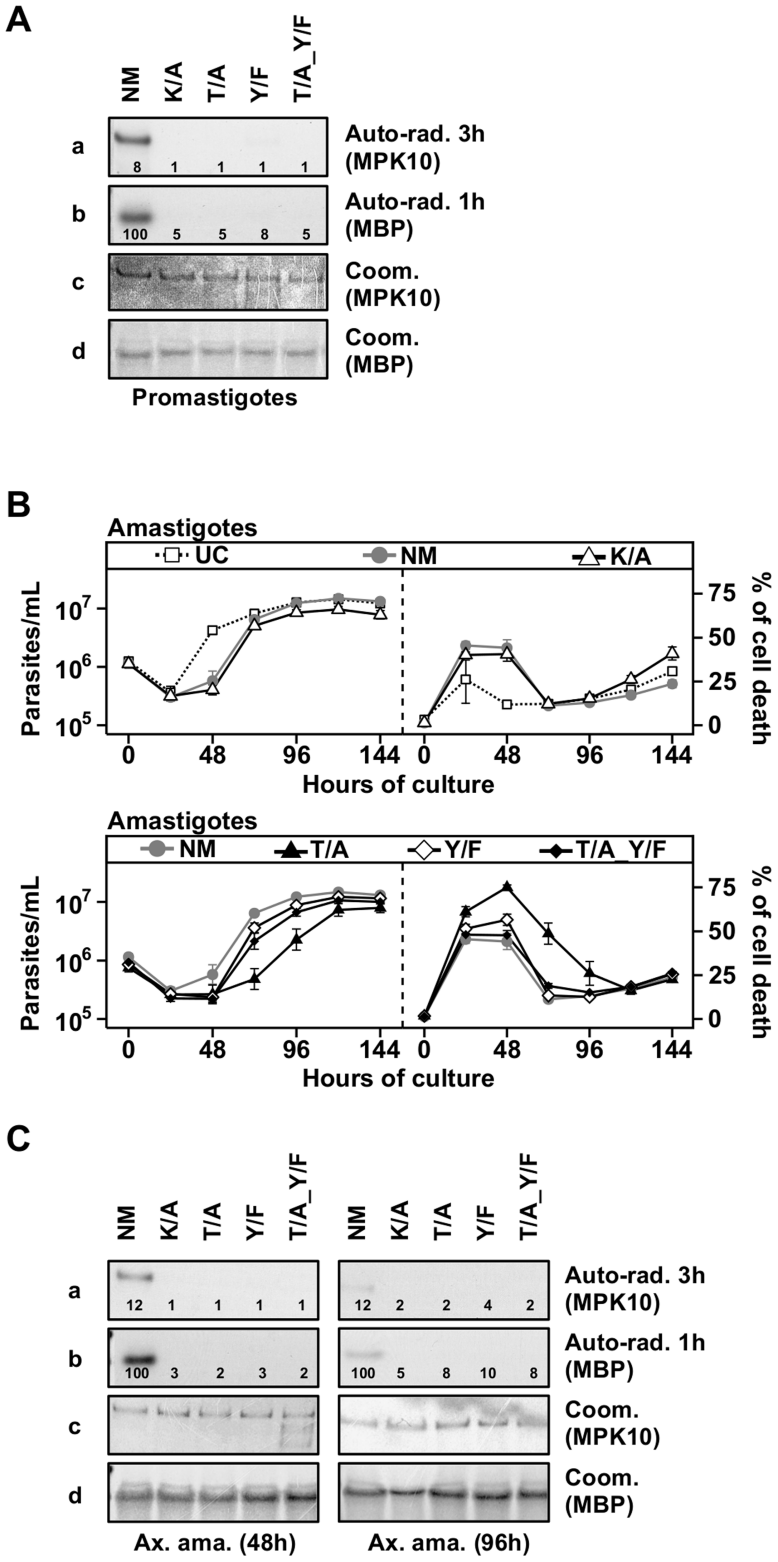
Both threonine 190 and tyrosine 192 are required for MPK10 activity. (**A**) *In vitro* kinase assay. Purified protein from promastigotes expressing non-mutated GFP-MPK10 (NM), and mutated GFP-MPK10-K51A (K/A), -T190A (T/A), -Y192F (Y/F) and -T190A_Y192F (T/A_Y/F) were incubated for 30 min at 37°C and pH 7.5 with 25 µg MBP as substrate. Reaction samples were separated by SDS-PAGE, gels were stained by Coomassie (Coom.), and signals revealed by auto-radiography (Auto-rad.) using the indicated exposure time. The numbers represent the level of radioactive counts as determined in a scintillation counter. The results were expressed relative to GFP-MPK10 NM from promastigotes of logarithmic culture set to 100%. Results are representative of three independent experiments. (**B**) Proliferation and viability analysis. 1×10^6^ amastigotes were cultured for 8 days and aliquots were taken every 24 h for analysis. Cell number and percent of cell death were assessed by flow cytometry. Upper panel: Untransfected Control, UC (dotted line, open squares); non-mutated GFP-MPK10, NM (grey line, grey circles); GFP-MPK10-K51A, K/A (black line, open triangles). Lower panel: GFP-MPK10-T190A, T/A (black line, black triangles); GFP-MPK10-Y192F, Y/F (black line, white diamonds); GFP-MPK10-T190A_Y192F, T/A_Y/F (black line, black diamonds). Mean values of the results obtained in two independent experiments in triplicate were plotted, with standard deviations indicated by the bars. (**C**) *In vitro* kinase assay. Non-mutated GFP-MPK10 (NM), and mutated GFP-MPK10-K51A (K/A), -T190A (T/A), -Y192F (Y/F) and -T190A_Y192F (T/A_Y/F) obtained from amastigotes at 48 h (left) and 96 h (right) during axenic differentiation were incubated for 30 min at 37°C and pH 7.5 with 25 µg MBP as substrate. Reaction samples were separated by SDS-PAGE, gels were stained by Coomassie (Coom.), and signals revealed by auto-radiography (Auto-rad.) using the indicated exposure time. The numbers represent the level of radioactive counts as determined by a scintillation counter. The results were expressed relative to GFP-MPK10 NM from promastigotes of logarithmic culture set to 100%. Results are representative of three independent experiments.

In most eukaryotes, both residues of the TxY motif need to be phosphorylated for MAPK activation and as a consequence their mutation abrogates kinase activity [41]–[43]. To analyze the requirement of T190 and Y192 phosphorylation for MPK10 activity, we used our transgenic cell lines to investigate the impact of the overexpression of the three different MPK10 TxY-motif mutants on parasite growth and survival as well as to measure MPK10 kinase activity, using GFP-MPK10 and GFP-MPK10-K51A as positive and negative controls, respectively. We first followed the growth and percentage of cell death in promastigotes by flow cytometry analysis and observed no differences between the untransfected *L. donovani* control (UC) and parasites over-expressing GFP-MPK10 NM or mutant forms (Figure S6). We next measured the kinase activity of GFP-MPK10 NM and GFP-MPK10 mutant proteins purified from promastigotes. The results presented in Figure 4A show that GFP-MPK10 NM undergoes weak auto-phosphorylation (exposure 3 h), but catalyzes robust MBP phosphorylation (exposure 1 h). It is interesting to note that although the auto-phosphorylation signals are much weaker than the MBP phosphorylation signals, they are similarly regulated. No MBP phosphorylation could be detected with the GFP-MPK10-K51A, -T190A, -Y192F or -T190A_Y192F mutants. These data demonstrate that, similarly to other eukaryotic MAPKs, T190 and Y192 are essential for MPK10 activity, as their mutation considerably reduced the activity of the kinase.

### Threonine 190 mutation affects axenic amastigote survival

We next investigated the impact of these MPK10 mutants on amastigote growth and survival. As presented in Figure 4B (upper panels), untransfected control (UC) parasites showed a decrease in cell growth during the first 24 hours of differentiation, but started to grow thereafter to reach a plateau at around 96 h. This profile was the consequence of a high level of cell death at 24 h (26±13%), which decreased to reach a percentage of cell death of 15±2% at 96 h. This phenomenon has been previously documented and is likely due to the adaptation of parasites to elevated temperature and acidic pH [44]. Parasites overexpressing GFP-MPK10 NM (Figure 4B, upper panels) resumed growth after 48 h of differentiation to finally reach the same cell concentration than UC parasites, a profile similar to that obtained with GFP-MPK10-K51A. We observed a higher percentage of cell death of GFP-MPK10 NM (41±5%) compared to UC parasites. This difference is largely attributed to the over-expression of MPK10, as there is only a slight yet statistically significant difference between the percentage of cell death of untransfected parasites and transgenic parasites expressing the empty vector during axenic amastigote differentiation (supplementary Figure S7). This finding, which was not observed in promastigotes, indicates that the over-expression of MPK10, regardless of its activity, is somewhat detrimental to axenic amastigotes.

We next compared the phenotype of strains over-expressing GFP-MPK10 to those over-expressing GFP-MPK10-Y192F, -T190A or -T190A_Y192F (Figure 4B, lower panels). All mutant strains showed a 24 hours growth delay, which corresponded to a higher percentage of cell death compared to that observed for GFP-MPK10 NM. At 24 h, strains expressing GFP-MPK10-Y192F and -T190A_Y192F showed a percentage of cell death of 51±7% and 48±6.1%, respectively (Figure 4B, lower panel right), but recovered thereafter and showed growth characteristics similar to the GFP-MPK10 NM used as control (Figure 4B, lower panel left). Parasites expressing GFP-MPK10-T190A showed a more severe phenotype with 61±7.6% of cell death (Figure 4B, lower panel right), from which they recovered slower than the other mutants (Figure 4B, lower panel left). This dominant negative effect, observed during the first 48 h after pH and temperature shift, corresponds to the period where MPK10 is the most active. Altogether, these data indicate that MPK10 could be important only transiently during differentiation. Moreover, these findings show that not only T190 phosphorylation is essential for the catalytic activity of MPK10 in promastigotes but also for axenic amastigote viability. Strikingly, increased cell survival can be restored in the GFP-MPK10-T190A mutant by Y192F mutation.

We then measured the kinase activity of the purified mutant proteins from parasites at 48 h of differentiation, when the percentage of cell death was the highest, and at 96 h after differentiation, when parasites have recovered. As shown in Figure 4C, we obtained similar results from amastigotes at 48 h (left panel) and 96 h (right panel) to those obtained from promastigotes, i.e. no kinase activity was detected for GFP-MPK10-K51A, -T190A, -Y192F or -T190A_Y192F. We did not find a clear correlation between over-expression phenotype and MPK10 kinase activity, as all mutant kinases were inactive, yet only the over-expression of GFP-MPK10-T190A was detrimental for the amastigotes, suggesting that the effect on growth is not entirely due to whether the protein is active or not. We only observed this phenotype in axenic amastigotes but never in promastigotes. This phenomenon may be due to the level of GFP-MPK10 expression, which was two- to five-fold lower in promastigotes compared to axenic amastigotes (data not shown). Thus, the level of transgenic MPK10 NM and MPK10 mutant protein could be too low to efficiently compete with the endogenous MPK10 in promastigotes, masking a dominant-negative effect. Why and how MPK10 levels are regulated in promastigotes and not in axenic amastigotes remains to be established. This observation seems independent from the vector, as expression of other kinases was not regulated the same way (data not shown). Altogether, these data confirm the essential role of both residues of the TxY motif for MPK10 activity.

### The C-terminal domain negatively regulates MPK10 activity *in situ*

**Figure 5:**
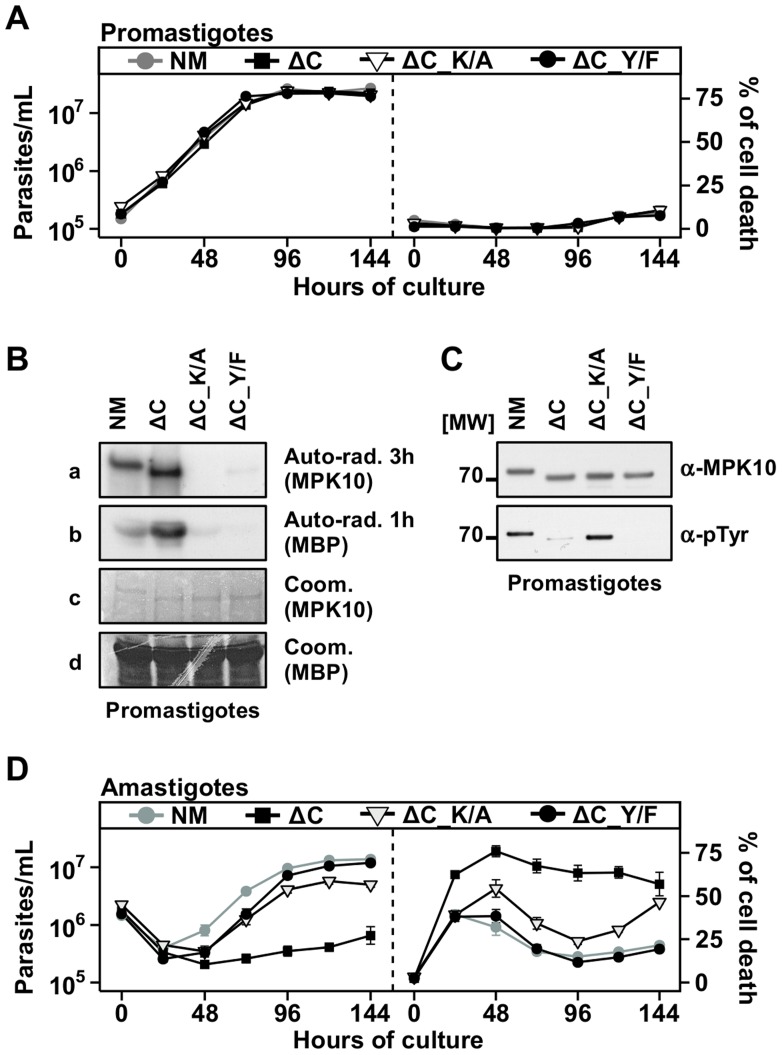
The C-terminal domain negatively regulates MPK10 activity *in situ*. (**A**), (**D**) Proliferation and viability analysis. The analyses represent the combined results of two triplicates experiments. 2×10^5^ promastigotes (A) and 1×10^6^ amastigotes (D) were cultured for 8 days and aliquots were taken every 24 h for analysis. Cell number and percent of cell death were assessed by flow cytometry. The cell lines that were tested are GFP-MPK10, NM (grey line, grey circles), GFP-MPK10-ΔC, ΔC (black line, black squares), GFP-MPK10-ΔC-K51A, ΔC-K/A (black line, open triangles), GFP-MPK10-ΔC-Y192F, ΔC-Y/F (black line, black circles). Mean values of the results obtained in two independent experiments in triplicate were plotted, with standard deviations indicated by the bars. (**B**) *In vitro* kinase assay. GFP-MPK10-ΔC_K51A (ΔC_K/A) and GFP-MPK10-ΔC_Y192F (ΔC_Y/F) obtained from respective transgenic promastigotes were incubated for 30 min at 37°C and pH 7.5 with 25 µg MBP as substrate. Reaction samples were separated by SDS-PAGE, gels were stained by Coomassie (Coom.), and signals revealed by auto-radiography (Auto-rad.) using the indicated exposure time. (**C**) Western blot analysis. Proteins were purified from promastigotes and analyzed by western blotting using anti-phospho-tyrosine (α-pTyr) and anti-MPK10 (α-MPK10) antibodies. All blots and auto-radiograms are representative of three independent experiments.

Our observation that auto-phosphorylation activity of recombinant MPK10 is enhanced after removal of the 46 C-terminal amino acids primed us to investigate the role of this domain in the regulation of MPK10 NM in its physiological context using our transgenic system. Compared to GFP-MPK10 NM transgenic parasites, GFP-MPK10-ΔC over-expression had neither an effect on promastigote growth (Figure 5A, left panel) nor cell death (Figure 5A, right panel). We next measured the kinase activity of this mutant. As judged by quantification using imageJ, a 3.5 fold increase in phosphorylation of MBP by GFP-MPK10-ΔC compared to that obtained by GFP-MPK10 was observed (Figure 5B), demonstrating that transgenic GFP-MPK10-ΔC has a higher activity than GFP-MPK10. Strikingly, GFP-MPK10-ΔC showed a strong increase of MBP phosphorylation but a moderate increase of auto-phosphorylation, suggesting that part of the increase in activity could be due to a better affinity for the substrate rather than enhanced phosphotransferase activity. This finding supports the hypothesis that the C-terminal domain has an auto-inhibitory function *in situ*. Surprisingly, Y192 phosphorylation was reduced by 80% in GFP-MPK10-ΔC compared to GFP-MPK10 NM (Figure 5C). Thus, although only 20% of GFP-MPK10-ΔC showed Y192 phosphorylation and thus can be considered active, the truncated kinase still phosphorylated MBP 3.5 fold more efficiently than GFP-MPK10 NM, suggesting a dramatic increase in kinase catalytic function after removal of the C-terminal domain.

We investigated in the following the effect of GFP-MPK10-ΔC over-expression on axenic amastigotes. Contrary to promastigotes, the over-expression of GFP-MPK10-ΔC caused an important reduction of cell growth after temperature and pH shift (Figure 5D, left panel), which resulted from an increase in the percentage of parasite death during the differentiation process, reaching 78±8.5% at 48 h. In contrast to the over-expression of the other mutants (Figure 4B), GFP-MPK10-ΔC transgenic parasites did not recover and maintained a high level of cell death. This result indicates that GFP-MPK10-ΔC is toxic for axenic amastigotes and as a consequence we were not able to investigate the kinase activity of GFP-MPK10-ΔC at this parasite stage. However, based on the enhanced activity detected in promastigotes, we hypothesized that the toxicity could be the consequence of a non-physiologically high level of MPK10 kinase activity. To test this possibility we generated two double mutants, GFP-MPK10-ΔC_K51A and -ΔC_Y192F that lack activity by different means. Over-expression of GFP-MPK10-ΔC_Y192F was no longer toxic for axenic amastigotes, suggesting that rendering GFP-MPK10-ΔC inactive is sufficient to rescue the parasites from the lethal phenotype as the percentage of cell death was significantly reduced compared to the active kinase (Figure 5D, right panel). GFP-MPK10-ΔC_K51A also showed a reduction in toxicity but not as complete as GFP-MPK10-ΔC_Y192F. This discrepancy was due to a higher percentage of cell death from 48 h to 144 h, which was not observed with the over-expression of GFP-MPK10-ΔC_Y192F. We next investigated whether this difference between GFP-MPK10-ΔC_K51A and -ΔC_Y192F could be explained by a difference in kinase activity but, as shown in Figure 5B and expected from the results obtained with the mutants of full length MPK10, both mutants showed a weak or no activity towards MBP, respectively. Thus, the different phenotypes observed with these two mutants are not linked to a difference in kinase activity. We further showed that in contrast to hyperactive GFP-MPK10-ΔC, the level of Y192 phosphorylation of inactive GFP-MPK10-ΔC_K51A corresponds to wild-type level (Figure 5C), thus revealing a negative feedback loop between MPK10 activity that controls the level of Y192 phosphorylation. Overall, these findings demonstrate that the toxicity following over-expression of GFP-MPK10-ΔC in amastigotes is due to the high activity of the truncated kinase, which can be compensated by reduction of tyrosine phosphorylation.

### Serine 395 could be implicated in MPK10 regulation

**Figure 6:**
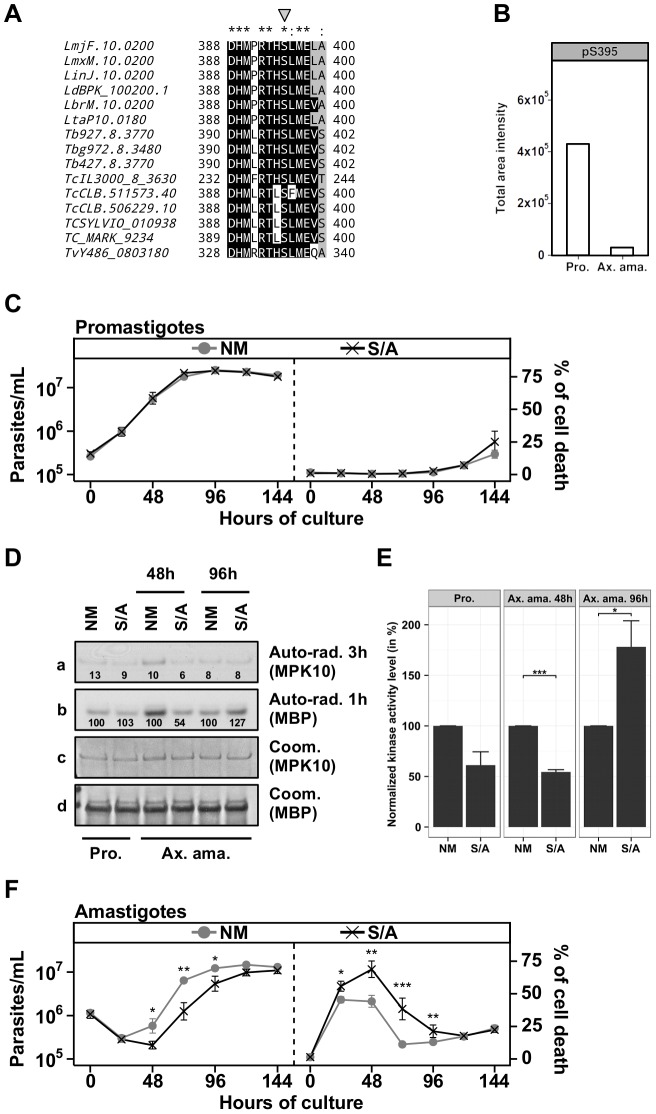
The regulatory Serine 395 residue shows stage-specific phosphorylation. (**A**) Multiple sequence alignment of MPK10 orthologs from trypanosomatids generated with Clustal-X and visualized with BioEdit. The phospho-serine residue S395 is marked by the grey arrow. LmjF, *L. major* Friedlin; LmxM, *L. mexicana* MHOM/GT2001/U1103; LinJ, *L. infantum* JPCM5; LdBPK, *L. donovani* BPK282A1; LbrM, *L. braziliensis* MHOM/BR/75/M2904; LtaP, *L. tarentolae* Parrot-TarlI; Tb, *T. brucei*; Tbg, *T. brucei gambiense* DAL972; TcIL3000, *T. congolense* IL3000; TcCLB, *T. cruzi* CL Brener; TCSYLVIO, T. *cruzi Sylvio* X10/1; Tc_MARK, *T. cruzi marinlellei* strain B7; TvY486, *T. vivax* Y486. Color code: Black, identical residues; Grey, similar residues; White, no conservation. (**B**) The level of phosphorylated S395 (pS395) isolated from promastigotes (P) and axenic amastigotes (Ax) was determined by SRM analysis as described in legend of Figure 3E. (**C**), (**F**) Proliferation and viability analysis. The analyses represent the combined results of two triplicates experiments. 2×10^5^ promastigotes (C) and 1×10^6^ amastigotes (F) were cultured for 8 days and aliquots were taken every 24 h for analyses. Cell number and percent of cell death were assessed by flow cytometry. The cell lines that were tested are GFP-MPK10 NM (grey line, grey circles), GFP-MPK10-S395A, S/A (black line, black crosses). Mean values of the results obtained in three independent triplicate experiments were plotted, with standard deviations indicated by the bars (*: p<0.05; **: p<0.01; ***: p<0.001). (**D**) *In vitro* kinase assay. Non-mutated GFP-MPK10 NM and mutated GFP-MPK10-S395A (S/A) obtained from promastigotes (Pro) and axenic amastigotes (Ax. Ama) at 48 h and 96 h during axenic differentiation were incubated for 30 min at 37°C and pH 7.5 with 25 µg MBP as substrate. Reaction samples were separated by SDS-PAGE, gels were stained by Coomassie (Coom.), and signals revealed by auto-radiography (Auto-rad.) using the indicated exposure time. The numbers represent the level of radioactive counts as determined by a scintillation counter. Signals were normalized to the counts obtained with GFP-MPK10 NM on MBP and set at 100%. (**E**) Histogram plot of the radioactive counts obtained from MBP after kinase assay normalized to the values obtained with GFP-MPK10 NM on MBP and set at 100%. Histograms represent mean values and the standard deviation is denoted by the error bars. Results are representative of three independent experiments. The asterisks represent statistical significance, with *: p<0.05; **: p<0.01; ***: p<0.001.

After demonstrating the importance of the C-terminal domain for auto-inhibition of MPK10 activity and parasite viability, we investigated the potential mechanisms involved in its regulation. Several mechanisms have been described to release kinases from auto-inhibition, including protein phosphorylation [45]. We performed a qualitative phospho-peptide analysis using *L. donovani* axenic amastigote extracts (48 h of axenic differentiation) to test this possibility. Our analysis identified the expected phospho-peptides encompassing the TxY motif (supplementary Figure S8A), but revealed a novel phospho-peptide located in the C-terminal domain of MPK10 showing a single phosphorylation at residue S395 (supplementary Figure S8B). This serine is conserved across MPK10 orthologs in trypanosomatids (Figure 6A, gray arrow), and part of the conserved sequence motif DHMxRTxSxME (underlined, Figure S3A), which suggests an important role for MPK10 function. If this serine were implicated in the regulation of MPK10 auto-inhibition, we would expect its phosphorylation to be stage regulated. To test this hypothesis we took advantage of the previously described quantitative phospho-peptide analysis. SRM analysis confirmed the presence of phosphorylated S395, which was mostly identified in promastigotes. Besides T190 of the activation loop this is the second stage-specifically regulated residue in MPK10 and thus may be crucial to control kinase activity (Figure 6B). As this promastigote-specific phosphorylation occurs when MPK10 is least active, dephosphorylation of S395 could be important to release MPK10 from the auto-inhibition.

We next investigated this possibility and studied the role of this residue in MPK10 regulation by generating transgenic parasites over-expressing GFP-MPK10-S395A. No difference was observed between GFP-MPK10 NM and -S395A with respect to promastigote growth (Figure 6C, left panel) or percentage of cell death (Figure 6C, right panel). Comparison of the kinase activities of GFP-MPK10 NM and -S395A purified from promastigotes did not reveal any difference based on quantification of the signals by a scintillation counter as detailed in Figure 3A (Figure 6D). We next compared the kinase activity of GFP-MPK10 NM and -S395A purified from parasites at different time points during axenic amastigote differentiation (Figure 6D). At 48 h, GFP-MPK10 NM phosphorylated MBP more efficiently than GFP-MPK10-S395A (100% and 54% respectively), whereas the activity of the mutant protein was slightly stronger than that of GFP-MPK10 NM at 96 h (127% and 100% respectively). These findings provide evidence for a delay in GFP-MPK10-S395A activation, supporting a role of this residue in proper kinase regulation.

The mean values with standard deviation of three independent experiments assessing activity for GFP-MPK10 NM and -S395A of parasites at 48 h and 96 h during axenic amastigote differentiation is shown in Figure 6E. Statistically significant differences were observed at 48 h with a reduction by twofold of GFP-MPK10-S395A activity (*p*-value<0.001), and at 96 h where on the contrary GFP-MPK10-S395A activity is increased by 75% (*p*-value<0.05). No significant difference was observed in promastigotes (*p*-value of 0.06). These data strongly suggest that in axenic amastigote, GFP-MPK10-S395A is differentially regulated compared to GFP-MPK10 NM, which could cause the observed effect on parasite viability. Parasites expressing GFP-MPK10-S395A presented a delay of 24 h in cell growth compared to GFP-MPK10 NM but thereafter resumed growth with kinetics slightly slower than that of parasites overexpressing GFP-MPK10 NM (Figure 6F, right panel). This delay is caused by a significantly higher percentage of cell death (69±16%, *p*-value<0.01) observed for parasites expressing GFP-MPK10-S395A at 48 h of axenic amastigote differentiation compared to GFP-MPK10 NM (44±12%, Figure 6F, right panel). In conclusion, our findings demonstrate the importance of S395 residue for axenic amastigote survival. The fact that the GFP-MPK10-S395A phenotype resembles that obtained with the over-expression of GFP-MPK10-T190A attributes similar importance for kinase regulation to S395 in the C-terminal domain as to T190 in the activation loop.

## Discussion

While the biological processes underlying *Leishmania* stage differentiation are poorly understood, the regulation of parasite development by environmental cues is firmly established. In eukaryotes, the MAPK pathway transmits environmental signals to trigger a wide range of cellular response. Thus studying the *Leishmania* MAPK pathway can provide new insight into molecular mechanisms underlying parasite differentiation and parasite-specific kinase biology. Here we uncover novel mechanisms regulating the *Leishmania* MAP kinase homolog MPK10 by utilizing two complementary approaches to study MPK10 regulation. First, recombinant expression and purification of MPK10 enabled us to identify a potential auto-inhibitory domain at the C-terminus of the protein. Second, transgenic expression and purification of MPK10 kinase allowed us (i) to validate MPK10 auto-inhibition *in situ*, (ii) to demonstrate the importance of the C-terminal domain for axenic amastigote survival, and (iii) to identify T190 and S395 as key regulatory residues. We propose that auto-inhibition of active MPK10 provides a fast signaling switch likely involved in environmentally induced pro- to axenic amastigote conversion and stage-specific regulation of MPK10 activity.

### MPK10 is an atypical eukaryotic MAPK

MAPKs are proline-directed serine/threonine kinases that phosphorylate substrates containing proline in the P+1 site [46]. Classically, MAPKs are inactive enzymes that are solely activated by M2Ks, which phosphorylate both the threonine and the tyrosine of the TxY motif present in the activation loop [9], [46]. This phosphorylation allows conformational changes that lead to the alignment of the R and C spines required for the activation of the kinase [29], [35], [36]. Moreover, the phosphorylation of the tyrosine residue of the TxY motif is important to permit the formation of the proline-directed P+1 specificity site required for substrate recognition and restriction of specificity [46]. The threonine residue possesses a structural role by stabilizing MAPK conformation and improving the geometry of the active site [46], [47]. MPK10 retains certain characteristics of classical MAPKs such as the conserved motif typical of MAPKs, TxYxxxRxYRxPE, including the TxY motif and the (P+1)-specificity pocket [16]. We demonstrated the importance of the T190 residue for the catalytic activity of MPK10, as alteration of this site abrogates phosphotransfer and severely reduces axenic amastigote survival. We also showed the essential role of the Y192 residue for MPK10 activity although its mutation does not have an impact on axenic amastigote survival unlike mutation of T190.

Aside these conserved MAPK features, MPK10 regulation presents many non-classical characteristics. We have previously shown that based on its structural conformation, MPK10 appears to be in an active conformation, without the need for dual-phosphorylation of the TxY motif, as the replacement of the DFG motif by a DFN motif results in the alignment of the R and C spines, similar to eukaryotic MAPKs after phosphorylation of the TxY motif by M2Ks [29]. Our study supports these findings revealing alternative modes of regulation of this intrinsic MPK10 activation state. We first showed that phosphorylation of Y192 is largely dissociated from MPK10 activity as its phosphorylation state does not show any significant stage-specific change, even though the activity of MPK10 increases by about twofold between log phase promastigotes and axenic amastigotes at 48 h after initiation of differentiation. In classical MAPKs, kinase activity correlates with phosphorylation of both the threonine and the tyrosine residues of the TxY motif, which is tightly regulated by environmentally induced upstream M3Ks and M2Ks [9]. By contrast, our data suggest that *Leishmania* MPK10 is mostly constitutively phosphorylated on Y192 in promastigotes and during amastigote development and proliferation. Consequently, this residue seems not to be implicated in regulating the observed stage-specific activation of MPK10. These findings were confirmed by proteomics identification of a mono-phosphorylated T190-H-pY192 activation loop in both pro- and axenic amastigotes, whereas dual pT190-H-pY192 phosphorylation of the activation loop was mainly identified in axenic amastigotes, where MPK10 is most active. However, we observed a decrease in Y192 phosphorylation in axenic amastigotes at stationary phase, which correlated with a decrease in MPK10 activity, suggesting that inactivation of MPK10 at this stage requires Y192 dephosphorylation.

Altogether, these findings indicate that the two residues of the TxY motif are differentially regulated, raising the question on how MPK10 is phosphorylated by M2Ks. There are at least five possibilities: (i) Y192 could be auto-phosphorylated in *cis*, a possibility that we rule out since kinase dead MPK10-K51A still shows phosphorylation on this residue. (ii) Classically, the phosphorylation of the TxY motif by M2Ks is sequential, initiating with the tyrosine residue [48]. M2Ks could constitutively phosphorylate the tyrosine residue of MPK10, but require additional signals or interactions to complete phosphorylation of the adjacent threonine residue. (iii) Each regulatory residue could be phosphorylated independently by two different M2Ks as it was demonstrated for human JNK kinase, whose threonine of the TxY motif is preferentially phosphorylated by MKK7, whereas its tyrosine is preferentially phosphorylated by MKK4 [9]. (iv) Without activation of the MAPK cascade, only Y192 could be accessible for phosphorylation, whereas access to T190 could be blocked by the C-terminal domain. (v) Both sites could be phosphorylated by the same M2K but only T190 would be constantly dephosphorylated until the MAPK pathway is fully activated. Three types of phosphatases target MAPKs, the dual-specificity phosphatases also called the MAP kinase phosphatases (MKPs), the tyrosine phosphatases such as PTP-SL and STEP, and finally the serine/threonine phosphatase such as PP2A [49]. PP2A could likely be responsible for this dephosphorylation; a possibility supported by recent findings that *T. cruzi* PP2A blocks axenic amastigote differentiation [50].

Dissociation of Y192 phosphorylation from stage-specific induction of MPK10 activation during amastigote differentiation raises the question of its importance for MPK10 function. We showed that similar to mammalian MAPKs, the Y192 residue is essential for MPK10 enzymatic activity. However, the dominant negative effect generated by overexpression of MPK10-T190A on axenic amastigote growth and viability was more detrimental than the one observed by overexpression of MPK10-Y192F, suggesting a more important role for the T190 residue for MPK10 activity. We can only speculate on the role of the largely constitutive phosphorylation of the Y192 residue. As described in the literature, phosphorylation of the tyrosine of the TxY motif is important to form the proline-directed (P+1)-specificity pocket [46], [49]. Consequently, maintaining Y192 mostly phosphorylated could allow a constant and dynamic interaction of MPK10 with its substrates allowing for faster phosphorylation once the pathway is activated.

A second striking feature of the regulation of MPK10 is represented by its C-terminal domain. Whether we used the recombinant or the transgenic protein, removal of this domain resulted in a significant increase in kinase activity. While this increase translates mainly into higher auto-phosphorylation levels for the recombinant kinase, we observed a dramatic increase in substrate phosphorylation for the transgenic kinase. One main difference between the two kinases is the absence of Y192 phosphorylation of the recombinant protein, which could explain the inability of recombinant kinase to efficiently transfer phosphate onto the substrate (Figure S5). Thus, our findings firmly establish regulation of MPK10 through auto-inhibition. This is a common feature in the regulation of kinase activities, and has been described for example for Twitchin kinase or CaMKI (Ca^2+^/calmodulin-dependent kinase I) but is a rare feature for MAPKs [48]. Although five human MAPKs have a long C-terminal domain (ERK3, ERK4, ERK5, ERK7 and ERK8), only that of ERK5 has an auto-inhibitory function [9]. The authors have shown that deletion of the last 100 amino acids of ERK5 leads to an increase of its kinase activity and they postulated that the deletion of the C-terminal domain could facilitate its activation by the upstream kinase [51]. Evidence for a different auto-regulatory mechanism of MPK10 arises from our analysis using transgenic parasites expressing the hyper-active MPK10 C-terminal deletion mutant: the strongly reduced phosphorylation levels of the regulatory Y residue in this mutant despite its significantly increased phosphotransferase activity demonstrates that the pool of active MPK10 is reduced, while the activity of each single active kinase protein is dramatically increased. Thus the deletion of the C-terminal domain increases the basal activity of the truncated kinase rather than allowing better access for the M2K. How the C-terminal domain regulates MPK10 activity is still elusive since no conserved pattern or domains such as SH2 or SH3 have been identified. One possible explanation could be that the C-terminal domain masks the active site acting as a pseudosubstrate such as described for Twitchin kinase [52]. It is interesting to note that in *T. brucei*, a hybrid kinase between a CDK (Cyclin dependent kinase) and a MAPK termed TbECK1 is also auto-inhibited by its C-terminal domain [32]. Procyclic parasites expressing the truncated protein showed growth defects and the parasites presented aberrant karyotypes. The expression of truncated TbECK1 was toxic to the bloodstream form, which is reminiscent to the toxic effect of MPK10-ΔC in axenic amastigotes.

Our data show that activating MPK10 via T190 is important for axenic amastigote survival, especially during the first 48 h after induction of axenic amastigote differentiation by temperature and pH shift. However, inhibition of MPK10 activity is equally essential as absence of auto-inhibition caused massive cell death in axenic amastigotes expressing MPK10-ΔC. These results therefore demonstrate the transient requirement for MPK10 during axenic amastigote differentiation and reveal a new mechanism to regulate MAPK activity by auto-inhibition, which is crucial for amastigote viability.

Finally, we identified a highly conserved, trypanosomatid-specific serine at position 395 inside the MPK10 C-terminal domain as a novel, stage-specific regulatory residue that is mainly phosphorylated in promastigotes and whose mutation causes an important dominant negative effect on axenic amastigote survival. Because kinases can be released from auto-inhibition by phosphorylation or dephosphorylation [45], we hypothesized that S395 could be implicated in the regulation of the auto-inhibition of MPK10 by its C-terminal domain. We did not confirm this hypothesis but we have two pieces of evidence suggesting that this residue could have an important role in the regulation of MPK10 activity. First, we showed that S395 is conserved in all trypanosomatids and is part of a sequence motif inside the C-terminal domain, which is also conserved in all trypanosomatids. Second, we clearly established the importance of the phosphorylation of S395 for axenic amastigote survival, which is as important as the regulation of T190, and partially mimics the viability defect caused by MPK10-ΔC. The function of this residue is still elusive and may be studied in the future by using an anti-phospho-S395 specific antibody to investigate the link between the phosphorylation kinetics of this residue and MPK10 activity, and address the question whether the release from auto-inhibition is regulated by environmental signals or whether it is alleviated before signal sensing, leaving the kinase in a semi-activated state, relying only on T190 phosphorylation for activation.

### MPK10 activity is regulated by a feedback loop

Because MPK10 shows a transient peak of activity during the first 48 h of axenic amastigote differentiation, its activity seems to be tightly regulated. The decrease in MPK10 activity after 48 h of induction of axenic amastigote differentiation is concomitant with the decrease of Y192 phosphorylation, suggesting that dephosphorylation of Y192 and probably T190 are required for MPK10 inactivation. We have evidence suggesting that MPK10 could directly or indirectly regulate its activity through the phosphorylation or the dephosphorylation of its TxY motif. Indeed, we have shown that to reduce toxicity caused by the over-expression of the hyperactive MPK10-ΔC, its level of Y192 phosphorylation was decreased. The level of pY192 was restored to wild-type level only after rendering the truncated kinase inactive, suggesting the existence of a feedback loop, where MPK10 could either inactivate the M2K that phosphorylates Y192 or activate the phosphatase that dephosphorylates this residue. This kind of feedback regulation between MAPKs and M2Ks or MKPs has not been extensively studied, as only three types of feedback loop have been described that are linked to regulation of MKPs, involving (i) rescue of MKP from degradation through phosphorylation by MAPK [53], (ii) activation of MKP by MAPK binding [54], and (iii) induction of MKP transcription by activated MAPK [55], [56], a possibility that can likely be discarded as transcription is constitutive in *Leishmania* [57]. In addition, Mody *et al*. have reported the phosphorylation of MKK5 by ERK5, revealing the existence of a potential feedback loop between a MAPK and its upstream activating kinase [58]. Future identification of the M2K and MKP that regulate the phosphorylation dynamics of the TxY motif of MPK10 will provide more insight into the mechanism controlling this feedback loop.

**Figure 7:**
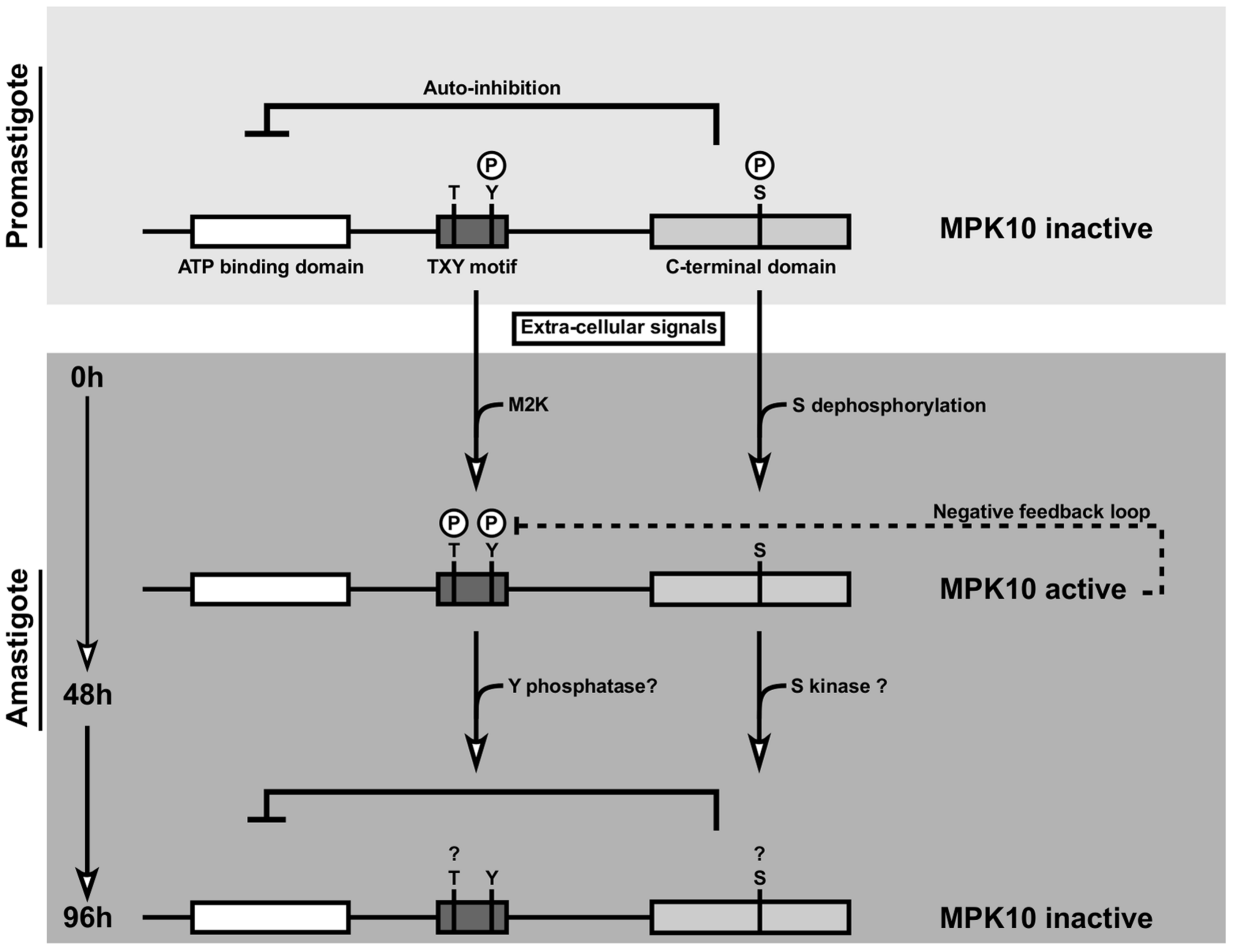
Model of MPK10 regulation based on our data. We propose a model in which MPK10 is not inactivated but partially active in promastigotes as judged by tyrosine phosphorylation and structural conformation. At this stage the kinase is kept in a standby configuration by auto-inhibition. During the first 48 h of axenic amastigote differentiation, MPK10 is released from auto-inhibition, which correlates with T190 phosphorylation and S395 dephosphorylation. This activity seems to be controlled by a feedback loop where MPK10 regulates its own tyrosine phosphorylation levels. Thereafter, MPK10 activity is decreased likely due to dephosphorylation of the TxY motif and phosphorylation of S395.

In conclusion, our transgenic study identifies novel mechanisms of MPK10 regulation that are unusual for MAPKs and document once more the stunning capacity of *Leishmania* to adapt highly conserved signaling proteins to its parasitic life style. Our data propose a model in which MPK10 is not inactivated but partially active in promastigotes as judged by tyrosine phosphorylation and structural conformation (Figure 7). At this stage the kinase is kept in a standby configuration by auto-inhibition. During the first 48 h of axenic amastigote differentiation, MPK10 is released from auto-inhibition, which correlates with T190 phosphorylation and S395 dephosphorylation. This activity seems to be controlled by a feedback loop where MPK10 regulates its own tyrosine phosphorylation levels. Thereafter, MPK10 activity is decreased likely due to dephosphorylation of the TxY motif and phosphorylation of S395. These regulatory residues may fine tune MPK10 regulation according to environmental signals and differentiation state through activating and inhibitory mechanisms. Future studies combining null mutant analysis and complementation assays for a detailed structure-function analysis of these residues and the auto-inhibitory C-terminal domain will uncover the contribution of these sequence elements in regulating MPK10 functions relevant for parasite differentiation and infectivity.

## Materials and Methods

### Parasite cell culture and differentiation

*Leishmania donovani* strain 1S2D (MHOM/SD/62/1S-CL2D), clone LdB was cultured and axenic amastigotes were differentiated as previously described [4] [59] [60]. Briefly, 10^6^ logarithmic promastigotes per mL were grown at 26°C in M199 media (supplemented with 10% heat-inactivated FCS, 25 mM HEPES pH 6.9, 4.2 mM NaHCO_3_ 7.5%, 2 mM glutamine, 8 µM 6-biopterin, 1× RPMI 1640 vitamin mix, 10 µg/mL folic acid, 100 µM adenine, 30 µM hemin, 100 U/mL of Penicillin/Streptomycin (Pen/Step)) and differentiated in axenic amastigotes by incubation at 37°C and 5% CO_2_ with RPMI media (supplemented with 20% of heat-inactivated FCS, 28 mM MES, 2 mM glutamine, 1× RPMI 1640 amino acid mix, 10 µM folic acid, 100 µM adenine, 100 U/mL of Pen/Step). Parasites were harvested at different time points between 12 h to 144 h after induction of axenic amastigote differentiation. For kinetic analysis, one flask (Corning) containing 200 mL of culture medium was used for each time point to avoid internal variations.

*Leishmania mexicana* MNYC/BZ/62/M379 promastigotes and axenic amastigotes were grown as described previously [61]. Cultures were incubated at 27°C until late-log phase (4–5×10^7^ parasites/ml), and either harvested or differentiated into amastigotes by inoculation in Schneider's Drosophila medium (PAN Biotech, Aidenbach, Germany) supplemented with 20% heat-inactivated FCS (PAN Biotech), 2 mM L-glutamine, 100 U/ml Pen/Strep, and 20 mM 2-morpholinoethanesulfonic acid monohydrate [MES] (Serva, Heidelberg, Germany) for a final pH of 5.5. Cultures were incubated at 34°C, 5% CO_2_, for 72 h. Parasites were harvested by centrifugation at 2,000× *g* at 4°C, and washed consecutively in ice-cold HEPES and ice-cold HEPES with protease and phosphatase inhibitors (1 mM Na-orthovanadate, 0.1 µM okadaic acid, 10 mM NaF, 10 mM *o*-phenanthroline, EDTA-free protease inhibitors (Roche)). Parasite pellets were snap-frozen in liquid nitrogen and stored at −80°C.

### Plasmids

#### pXG-GFPK10

For transgenic expression of GFP-MPK10 fusion protein, the gene encoding the *L. major* MAPK homologue LmaMPK10 (accession number CAJ02415) was PCR amplified from 50 ng of genomic DNA of *L. major* FVI using primers 5′-ACCAGATCTCCACCATGCAGGCCAAGGGC-3′ (forward) and 5′-GCGAGATCTTCACGACGGGGCCGGCGC-3′ (reverse) and cloned into pGEM-T (Promega) to obtain pGEMT-MPK10. This plasmid was subsequently digested by BglII and subcloned into pXG-GFP+2 to generate pXG-GFP-MPK10 (kindly provided by S. Beverley, Washington University Medical School, St. Louis, MO, USA). The various MPK10 mutants described in the results section were obtained by site direct mutagenesis of pGEMT-MPK10 using the QuickChange II XL Site-Directed Mutagenesis kit (Stratagene) prior to subcloning into pXG-GFP2+. MPK10-ΔC was PCR amplified with primers 5′-ACCAGATCTCCACCATGCAGGCCAAGGGC-3′ (forward) and 5′-GCGAGATCTTCAGTCGTTGAAGCGCTCC-3′ (reverse) containing a stop codon to generate a protein deleted for the last 46 amino-acids (138 nucleotides) from the C-terminal end of LmaMPK10, and was cloned into pXG-GFP2+ as described above to generate the pXG-GFP-MPK10-ΔC construct. All constructs were validated by sequence analysis.

#### pQE80-His_6_-MPK10

For recombinant protein production, the sequences encoding for LmaMPK10 and LmaMPK10-ΔC were amplified by PCR and subcloned into plasmid pQE80 (Qiagen, Venlo, the Netherlands), previously modified to carry a Tobacco Etch Virus (TEV) protease cleavage site for removal of the N-terminal His-tag as previously described [29]. The same strategy as described above was applied to obtain mutated kinase constructs. All constructs were validated by sequence analysis.

#### pGEX-GST-Strep3-MPK10

An alternative expression construct for recombinant MPK10 production was created by PCR amplification of LmaMPK10 with primers 5′-TTCGAAAAAGGTGGAATGCAGGCCAAGGGCG-3′ (forward) and 5′-CCCGGGTCACGACGGGGCCGGCG-3′ (reverse), and the fragment was cloned into pGEM-T vector to obtain pGEM-MPK10-NcoI-BstBI. Primers 5′-CATGGATCCTTATGTGGAGCCACCCGCAGTTCGAGAAAGGTGGAGGTTCCTGGAGCCACCCGCAGTT-3′ and 5′-CGAACTGCGGGTGGCTCCAGGAACCTCCACCTTTCTCGAACTGCGGGTGGCTCCACATAAGGATC-3′ were annealed and subsequently phosphorylated to generate a double stranded oligonucleotide containing the Strep3 sequence with 5′ overhangs. This fragment was cloned into pGEM-MPK10, digested by NcoI/BstBI, to generate the pGEM-Strep3-MPK10 plasmid. pGEM-Strep3-MPK10 was digested by BamHI/NotI and cloned into pGEX-5X-1 (Abbgene) to generate pGEX-GST-Strep3-MPK10. The same strategy as described above was applied to obtain mutated kinase constructs. All constructs were validated by restriction enzyme digestion and sequencing.

### Parasite transfection

Episomal tranfectants were generated by electroporation of 5×10^7^ *L. donovani* LdB promastigotes from logarithmic culture with 20 µg of plasmid [62]. Transfected cells were selected in liquid media containing 20 µg/ml geneticin (Invitrogen) and resistant parasites were expanded in liquid culture at drug concentrations up of to 100 µg/ml of geneticin. Parasites were then frozen one passage after selection and all experiments were performed with parasites issued from the same electroporation. To avoid any potential bias due to adaptation or compensatory responses, parasites were used for all the experiments at passage 2 after selection. Pools of transfectants were used to avoid clonal variation that can bias transgenic studies.

### Cell death analysis

Cultured parasites were incubated for 15 min with 10 µg/ml propidium iodide (Sigma-Aldrich) and diluted in PBS (Gibco). Cells were analyzed with a FACSCalibur flow cytometer (Beckman Coulter) to determine the incorporation of propidium iodide (excitation wave length λ_ex_ = 488 nm; emission wave length λ_em_ = 617 nm). The percentages of cell death and cell growth were calculated using FlowJo (v7.6) software (Tree Star, Inc., San Carlos, CA).

### Protein extraction and purification

10^9^ parasites were washed with ice cold RPMI and lysed in 1 ml lysis buffer containing 150 mM NaCl, 1% Triton X-100, 50 mM Tris HCl pH 8 and inhibitor cocktails for proteases (Complete Mini EDTA-free tablets, Roche Applied Science, IN) and phosphatases (Phosphatase Inhibitor Cocktails I and II, Sigma–Aldrich, MO). Clear lysates were obtained after sonication and centrifugation at 12 000× *g* for 10 min and stored at −80°C.

Purified GFP-MPK10 wild-type and mutant proteins were isolated from crude cell extracts of respective transgenic parasites using the μMACS Epitope Tag Protein Isolation Kit, according to the manufacturer's specifications (Miltenyi Biotec Inc., CA). Briefly, equal amounts of total proteins were incubated with 50 µl of magnetic bead-conjugated mouse monoclonal anti-GFP antibody for 1 hour at 4°C, immuno-complexes were immobilized on the μMACS separator, washed four times with 150 mM NaCl, 1% Igepal CA-630 (formerly NP-40), 0.5% sodium deoxycholate, 0.1% SDS, 50 mM Tris HCl (pH 8.0), and once with 20 mM Tris HCl (pH 7.5). Bound GFP-MPK10 protein was eluted in 75 µl PBS after removing the columns from the magnetic field.

### SDS-PAGE and western blot analysis

Purified proteins were separated by SDS–PAGE (NuPAGE gel 4–12% Bis-Tris, Invitrogen) and visualized either by Coomassie staining or SYPRO Ruby Protein Gel Stain (Invitrogen) using a Typhoon 9400 scanner (Amersham Biosciences) with λ_ex_ = 457 nm and λ_em_ = 610 nm. Alternatively, proteins were separated by SDS–PAGE on NuPAGE 4–12% Bis-Tris gels (Invitrogen) and blotted onto polyvinylidene difluoride (PVDF) membranes (Pierce). Proteins were revealed using the following antibodies at the indicated dilutions: i) polyclonal anti-MPK10 antibody, generated by rabbit immunization using recombinant MPK10 protein produced in *E. coli* transformed with pGEX-Strep3-MPK10 plasmid (Eurogentec), 1∶10,000; ii) anti-phospho-tyrosine antibody 4G10 Platinum from Millipore, 1∶1,000; and iii) secondary goat anti-rabbit-HRP and anti-mouse-HRP antibodies from Thermo Scientific, 1∶20,000. The visualization was performed on X-ray film (Roche) at various exposure times.

### Recombinant expression and purification of MPK10

*E. coli* BL21 Rosetta (VWR) transformed with pQE80-His_6_-MPK10 or pGEX-GST-Strep3-MPK10 were grown at 37°C and induced with IPTG (0.2 µM final) overnight at RT. Cells were harvested by centrifugation at 15,000× *g* for 10 min at 4°C.

For the purification of GST-Strep3-MPK10, bacteria were resuspended in a pre-chilled buffer containing 25 mM Tris-HCl pH 8, 150 mM NaCl, 1 mM DTT, 10 µg/mL apoprotein, 1 µM leupeptin, 1 µM pepstatin, 1 mM PMSF. Samples were sonicated (Bioruptor system, Diagenode) for 2 min at 20 V setting on ice (10 s on/10 s off cycle). After addition of 500 µg/mL of lysozyme and 500 U of benzonase, lysates were incubated on ice for 30 min with 140 µM EDTA, 0.0035% Triton-X100 and centrifuged at 15,000× *g* for 30 min at 4°C. The supernatant was immediately subjected to GST affinity chromatography (GSTrap, GE Healthcare Life Sciences, Waukesha, WI, USA). GST-tagged proteins were washed with a buffer containing 50 mM Tris pH 8, 100 mM NaCl, 1 mM DTT and eluted with the same buffer supplemented with 30 mM L-glutathione. Appropriate fractions were pooled and the GST-tag was cleaved by incubation of the fractions with 5 µg/mL Xa factor in presence of 1 mM CaCl_2_. The reaction was stopped by adding 5 µg/mL of Glutamyl-glycyl-arginine chloromethyl ketone GGACK (Calbiochem). A second purification step was performed using a StrepTrap column (Strep-tactin, GE Healthcare Life Sciences, Waukesha, WI, USA). Elution was performed with E Strep buffer (100 mM Tris-HCl pH 8, 150 mM NaCl, 1 mM EDTA and 2.5 mM Desthiobiotin) and appropriate fractions were collected, and stored at 4°C until used.

For His_6_-MPK10 purification, the bacterial pellet was resuspended in PBS containing 60 mM β-glycerophosphate, 1 mM sodium vanadate, 1 mM sodium fluoride, 1 mM disodium phenylphosphate, 150 mM sodium chloride, 10 mM imidazole supplemented with protease inhibitor cocktail (Complete EDTA free tablets, Roche Applied Science). The sample was sonicated for 2 min at 20 V setting on ice (10 s on/10 s off cycle). Triton X-100 (0.1% final) was added, the sample was incubated for 30 min at 4°C (shaking) and centrifuged at 15,000× *g* for 30 min at 4°C. The supernatant was purified on Co-NTA agarose (Pierce). The beads were washed with PBS containing 60 mM β-glycerophosphate, 1 mM sodium vanadate, 1 mM sodium fluoride, 1 mM disodium phenylphosphate, 300 mM sodium chloride, 30 mM imidazole, 1% Triton X-100 at pH 7.5. Elution was performed with 300 mM imidazole in elution buffer pH 7.5 (PBS containing 60 mM β-glycerophosphate, 1 mM sodium vanadate, 1 mM sodium fluoride, 1 mM disodium phenylphosphate). The eluate was supplemented with 15% glycerol and stored at −80°C.

### Kinase assay

Ten percent of the GFP-MPK10 purified protein was incubated on a shaker for 30 min at 37°C with 25 µg myelin basic protein (MBP) substrate, 200 µM of ATP, 50 mM of MOPS pH 7.5, 100 mM NaCl, 10 mM MgCl_2_ and 1 µCi [γ-^32^P] adenosine-triphosphate (ATP) (3000 Ci/mmol) in final volume of 20 µl. The phosphotransferase reaction was then stopped by adding Laemmli loading buffer. Reaction mixtures were separated by SDS–PAGE, which was stained by Commassie and dried. ^32^P incorporation was monitored by exposing the dried gel on an X-ray sensitive film (Roche) at −80°C. After exposure, the bands corresponding to MPK10 or MBP were excised from dried gels and radioactivity was quantified by a scintillation counter.

Recombinant His_6_-MPK10 and respective mutants were assayed with 36 µg dephosphorylated casein, 12 µg histone H1, 9 µg of Ets1 or 9 µg MBP as substrates in a Tris buffer at pH 7.5 (50 mM Tris-Cl pH 7.5, 10 mM MnCl_2_ and 100 mM NaCl) in 20 µl final volume and in the presence of 15 µM [γ-^33^P]-ATP. After 30 min incubation at 37°C, the reaction was stopped by adding an equal volume of 2× electrophoresis loading buffer to the 20 µl reaction mix. Incorporated ^33^P was monitored by auto-radiography.

### Partial tryptic digestion of recombinant MPK10

50 µg of Strep3-MPK10 were digested with 0.25 µg trypsin at RT. Aliquots were taken at 0, 2.5, 5, 15, 30, 60, and 150 min and the reaction was stopped by adding Laemmli loading buffer. The polypeptides were then separated by SDS-PAGE, transferred to PVDF membrane, stained with amidoblack, and N-terminal sequencing was performed. For the mass determination of cleavage products, pH of the cleavage reaction was lowered to 5.0 and mass determination was performed by SELDI-TOF analysis after immobilizing the samples on a H4 ProteinChip Array (C16 reversed phase surface).

### Qualitative phosphopeptides determination

A *Leishmania* amastigote cell pellet was submitted for qualitative phosphoproteomic analysis using titanium dioxide phosphopeptide enrichment followed by an iTRAQ labeling experiment for analysis of phosphopeptides by LC-MS/MS. Briefly, 400 µg of *Leishmania* proteins were reduced with DTT and the free cysteines were alkylated with iodoacetamide for 30 min at 37°C in darkness. Proteins were then digested overnight at 37°C using Porcine trypsin (sample:enzyme ratio of 50∶1). Following the digestion, the peptides were acidified, concentrated and de-salted using a Waters HLB Oasis SPE cartridge. The peptides were then enriched for phosphopepitdes using a TiO_2_ affinity column and splited into two 100 µL aliquots. Each aliquot was then reduced and alkylated with MMTS followed by iTRAQ labeling (114, 116). The 116 labeled sample was then treated with FastAPalkaline phosphatase before the samples were combined, fractionated by SCX chromatography and analyzed by ESI-Q-TOF MS/MS. Sample were then analyzed by reversed phase nanoflow (300 nL/min) HPLC with nano-electrospray ionization using a quadrupole time-of-flight mass spectrometer (QSTAR Pulsar I, Applied Biosystems) operated in positive ion mode and a 2 hour gradient.

### Quantitative phosphopeptide identification and quantification

1×10^7^ parasites were lysed in a solution of 7 M urea, 2 M thiourea, 40 mM Tris, 1% n-octyl-β-D-glycopyranoside, 1 mM MgCl_2_, 1 mM o-phenanthroline, 300 U benzonase, 1 mM Na-pervanadate (Na-orthovanadate activated in 18% H_2_O_2_), EDTA-free protease inhibitors (Roche) and phosphatase inhibitor cocktails (P2850 and P5726 from Sigma), and sonicated for 3×15 s on ice. Lysates were incubated at −80°C for 30 min prior to reduction (DTT, 20 mM, incubation at room temperature for 60 min) and alkylation (iodoacetamide, 40 mM, incubation at room temperature in the dark for 45 min). Proteins were precipitated in 8 fold excess of ice-cold acetone-ethanol (1∶1, v/v) by overnight incubation at −20°C. Proteins were reconstituted in 6 M urea/2 M thiourea and diluted in 50 mM NH_4_HCO_3_ for digestion with trypsin at a 75∶1 substrate-enzyme ratio over night. For selected reaction monitoring (SRM) analyses, 3×330 µg digested protein from whole cell lysates of promastigotes, axenic amastigotes were subjected to TiO_2_ enrichment as described in Rosenqvist et al. [40]. The TiO_2_ eluates were pooled prior to SRM analysis. The discovery analyses were conducted in triplicates for each of the sample types using LTQ Orbitrap XL mass spectrometers (Thermo Fisher Scientific, Bremen, Germany). The discovery data were processed with DTASuperCharge (Mortensen, P. DTASuperCharge, an MSQuant application. http://msquant.alwaysdata.net/msq/) and searched against a customized *L. mexicana* 6-frame translation library as well as a predicted protein list (GeneDB http://www.genedb.org) using an in-house Mascot server (version 2.2.06, Matrix Science, London, UK). For SRM analyses, the discovery data were processed in ProteomeDiscoverer, and the MPK10 phosphopeptides identified were imported into Pinpoint, version 1.0.0 (Thermo Scientific).

### Bioinformatics approaches

*Leishmania spp* and *Trypanosoma spp* MPK10 gene and protein sequences were retrieved from the web databases GeneDB (www.genedb.org) and TriTrypDB (http://tritrypdb.org/tritrypdb/) [63]. Homology searches were carried out using BLAST Program with the default BLOSUM-62 substitution matrix [64], and pattern recognition analysis using the program PRATT v2.1 [65]. Multiple sequence alignments were performed using built-in algorithm ClustalXv2. Additional sequence analyses were carried out using the BioEdit Program suite (Tom Hall, North Carolina State University). Statistical analysis and data plotting were performed using Rstudio software (http://www.rstudio.org/) and R language (R Development Core Team (2005). R: A language and environment for statistical computing. R Foundation for Statistical Computing, Vienna, Austria. ISBN 3-900051-07-0, URL: http://www.R-project.org). Statistical analyses were performed using the t-test on mean values when samples followed a Normal distribution, otherwise the Mann-Whitney Rank Sum Test was used. Differences were considered significant when p value<0.05.

## Supporting Information

Figure S1 A) Multiple sequence alignment of MPK10 orthologs from *Leishmania* generated with Clustal-X and visualized with BioEdit. Color code: black, identical residues; grey, similar residues; white, no conservation. B) Table of percent of protein sequence identity of MPK10 from *L. donovani* compared to other *Leishmania* species. LmjF, *L. major* Friedlin; LmxM, *L. mexicana* MHOM/GT2001/U1103; LinJ, *L. infantum* JPCM5; LdBPK, *L. donovani* BPK282A1; LbrM, *L. braziliensis* MHOM/BR/75/M2904; LtaP, *L. tarentolae* Parrot-TarlI.
(TIF)

Figure S2 Effect of pH variation on the activity of recombinant MPK10. Non-mutated GST-Strep-MPK10 NM and kinase dead mutant K/A were incubated with casein for 30 min at pH 5.5, 6.5, 7.5 and 8.5 at 37°C. Recombinant human MEK1 was used as positive control with MBP as substrate at pH 7.5. MW, Molecular Weight.
(TIF)

Figure S3 A) Multiple sequence alignment of the C-terminal domain of MPK10 orthologs from *Trypanosomatidae* generated with Clustal-X and visualized with BioEdit. The phospho-serine residue S395 is marked by the grey arrow and the conserved DHMxRTxSxME motif is underlined. Color code: black, identical residues; grey, similar residues; white, no conservation. B) Table of percentage of protein sequence identity of MPK10 from *L. donovani* compared to other *Leishmania* species. The following strains were aligned LmjF, *L. major* Friedlin; LmxM, *L. mexicana* MHOM/GT2001/U1103; LinJ, *L. infantum* JPCM5; LdBPK, *L. donovani* BPK282A1; LbrM, *L. braziliensis* MHOM/BR/75/M2904; LtaP, *L. tarentolae* Parrot-TarlI; Tb, *T. brucei*; Tbg, *T. brucei gambiense* DAL972; TcIL3000, *T. congolense* IL3000; TcCLB, *T. cruzi* CL Brener; TCSYLVIO, T. *cruzi Sylvio* X10/1; Tc_MARK, *T. cruzi marinlellei* strain B7; TvY486, *T. vivax* Y486.
(TIF)

Figure S4 Table of the *p*-values for the kinetic analysis of kinase activity plotted in Figure 3B. Results of three independent experiments were subjected to statistical analysis using a student's t-test or Mann-Whitney Sum Rank test. Equal gray level of the cells corresponds to equal *p*-value. Dark grey, *p*-value<0.001; light grey, *p*-value<0.01; white, *p*-value<0.05, NS, non significant.
(TIF)

Figure S5 Recombinant MPK10 is not phosphorylated on Y192. GFP-MPK10 and GST-Strep-MPK10 NM and -K/A proteins were purified from amastigotes at 48 h during axenic differentiation and recombinant bacteria, respectively, and analyzed by western blotting using anti-phospho-tyrosine (α-pTyr) and anti-MPK10 (α-MPK10) antibodies.
(TIF)

Figure S6 MPK10 mutations do not affect promastigote growth and viability. The analysis represents the combined results of three triplicate experiments. 2×10^5^ promastigotes were cultured during 8 days and aliquots were taken every 24 h for analysis. Cell number and percent of cell death were assessed by flow cytometry. Upper panel: Untransfected Control, UC (dotted line, open squares); GFP-MPK10, NM (grey line, grey circles); GFP-MPK10-K51A, K/A (black line, open triangles). Lower panel: GFP-MPK10-T190A, T/A (black line, black triangles); GFP-MPK10-Y192F, Y/F (black line, white diamond); GFP-MPK10-T190A_Y192F, T/A_Y/F (black line, black diamond). Each point of the line graph represents the mean of three independent triplicate experiments, with standard deviations denoted by error bars.
(TIF)

Figure S7 Overexpression of empty vector has only a slight effect on amastigote viability. The analysis represents one triplicate experiment. 1×10^6^ amastigotes were cultured during 8 days and aliquots were taken every 24 h for analysis. Cell number and percent of cell death were assessed by flow cytometry. Upper panel: Untransfected Control, UC (dotted line, open squares); GFP-MPK10, NM (grey line, grey circles); pXG-mock, mock (black line, black circles). Each point of the line graph represents the mean of three independent triplicate experiments, with standard deviations denoted by error bars.
(TIF)

Figure S8 LC-MS/MS analysis of *Leishmania* total protein extracts from amastigotes. The samples were subjected to phosphoproteomic analysis following titanium dioxide phosphopeptide enrichment and iTRAQ labeling. MS/MS raw data spectra of identified peptides presenting phosphorylation of threonine and tyrosine of the TxY motif (A), and phosphorylation of serine 395 located inside the carboxy terminal extension of MPK10 (B) are shown.
(TIF)
