# Supplementary figures and images for: Transgenic Analysis of the Leishmania MAP Kinase MPK10 Reveals an Auto-inhibitory Mechanism Crucial for Stage-Regulated Activity and Parasite Viability
Source: PLoS Pathog. 2014 Sep 18;10(9):e1004347. doi: 10.1371/journal.ppat.1004347 (PMC4169501; doi:10.1371/journal.ppat.1004347)

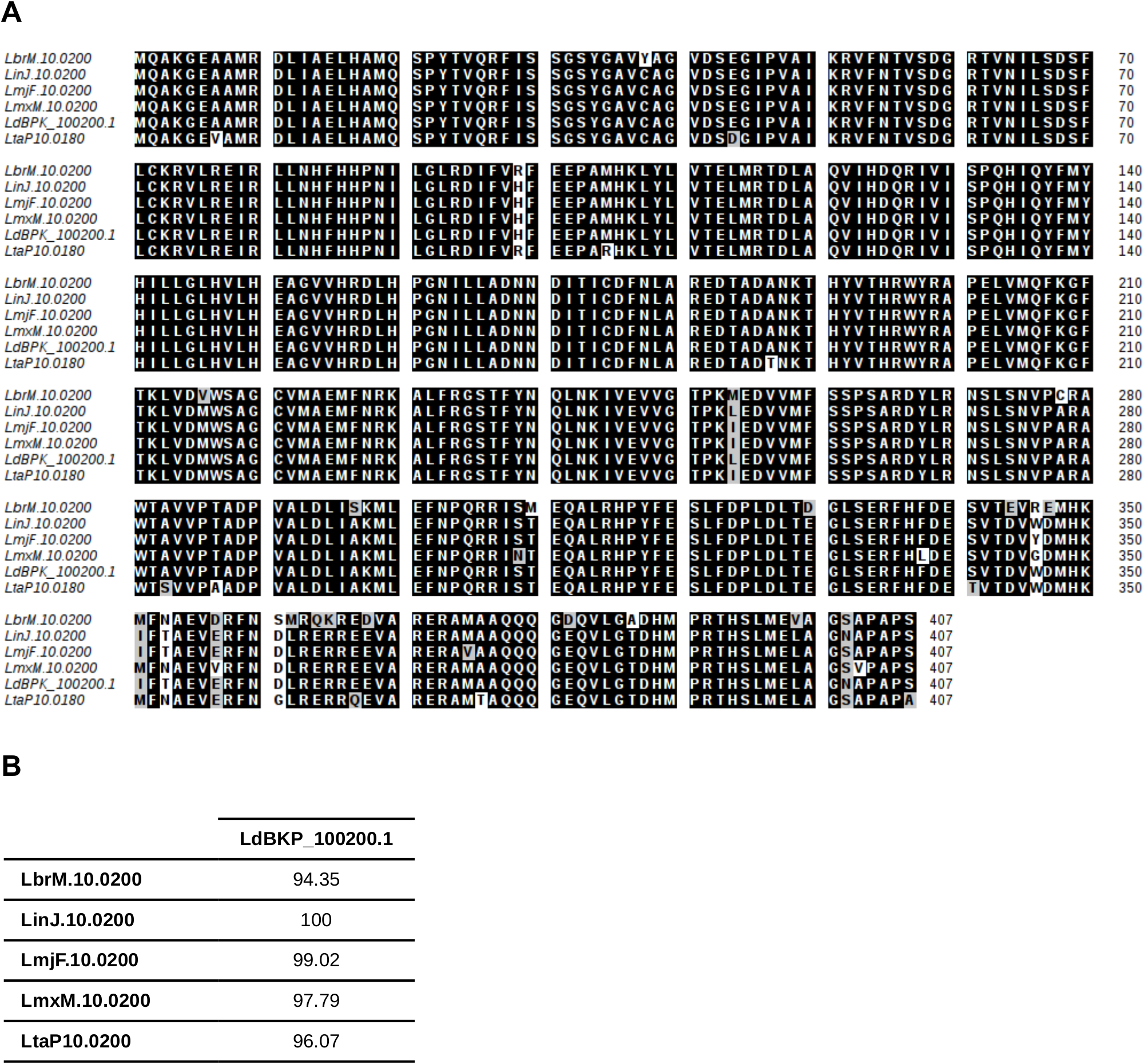

Supplement: Figure S1 — A) Multiple sequence alignment of MPK10 orthologs from Leishmania generated with Clustal-X and visualized with BioEdit. Color code: black, identical residues; grey, similar residues; white, no conservation. B) Table of percent of protein sequence identity of MPK10 from L. donovani compared to other Leishmania species. LmjF, L. major Friedlin; LmxM, L. mexicana MHOM/GT2001/U1103; LinJ, L. infantum JPCM5; LdBPK, L. donovani BPK282A1; LbrM, L. braziliensis MHOM/BR/75/M2904; LtaP, L. tarentolae Parrot-TarlI. (TIF) [file ppat.1004347.s001.tif]

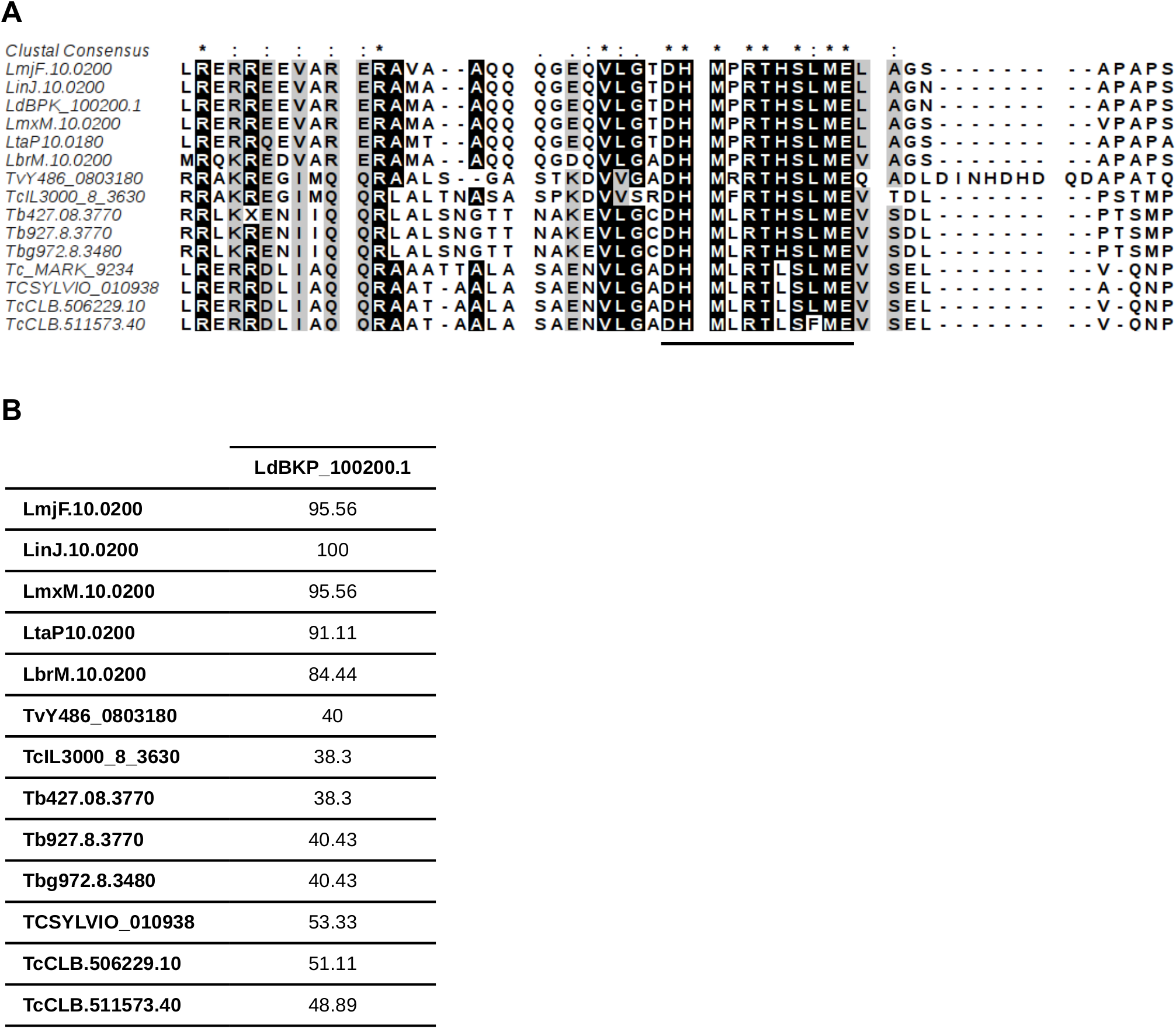

Supplement: Figure S3 — A) Multiple sequence alignment of the C-terminal domain of MPK10 orthologs from Trypanosomatidae generated with Clustal-X and visualized with BioEdit. The phospho-serine residue S395 is marked by the grey arrow and the conserved DHMxRTxSxME motif is underlined. Color code: black, identical residues; grey, similar residues; white, no conservation. B) Table of percentage of protein sequence identity of MPK10 from L. donovani compared to other Leishmania species. The following strains were aligned LmjF, L. major Friedlin; LmxM, L. mexicana MHOM/GT2001/U1103; LinJ, L. infantum JPCM5; LdBPK, L. donovani BPK282A1; LbrM, L. braziliensis MHOM/BR/75/M2904; LtaP, L. tarentolae Parrot-TarlI; Tb, T. brucei; Tbg, T. brucei gambiense DAL972; TcIL3000, T. congolense IL3000; TcCLB, T. cruzi CL Brener; TCSYLVIO, T. cruzi Sylvio X10/1; Tc_MARK, T. cruzi marinlellei strain B7; TvY486, T. vivax Y486. (TIF) [file ppat.1004347.s003.tif]

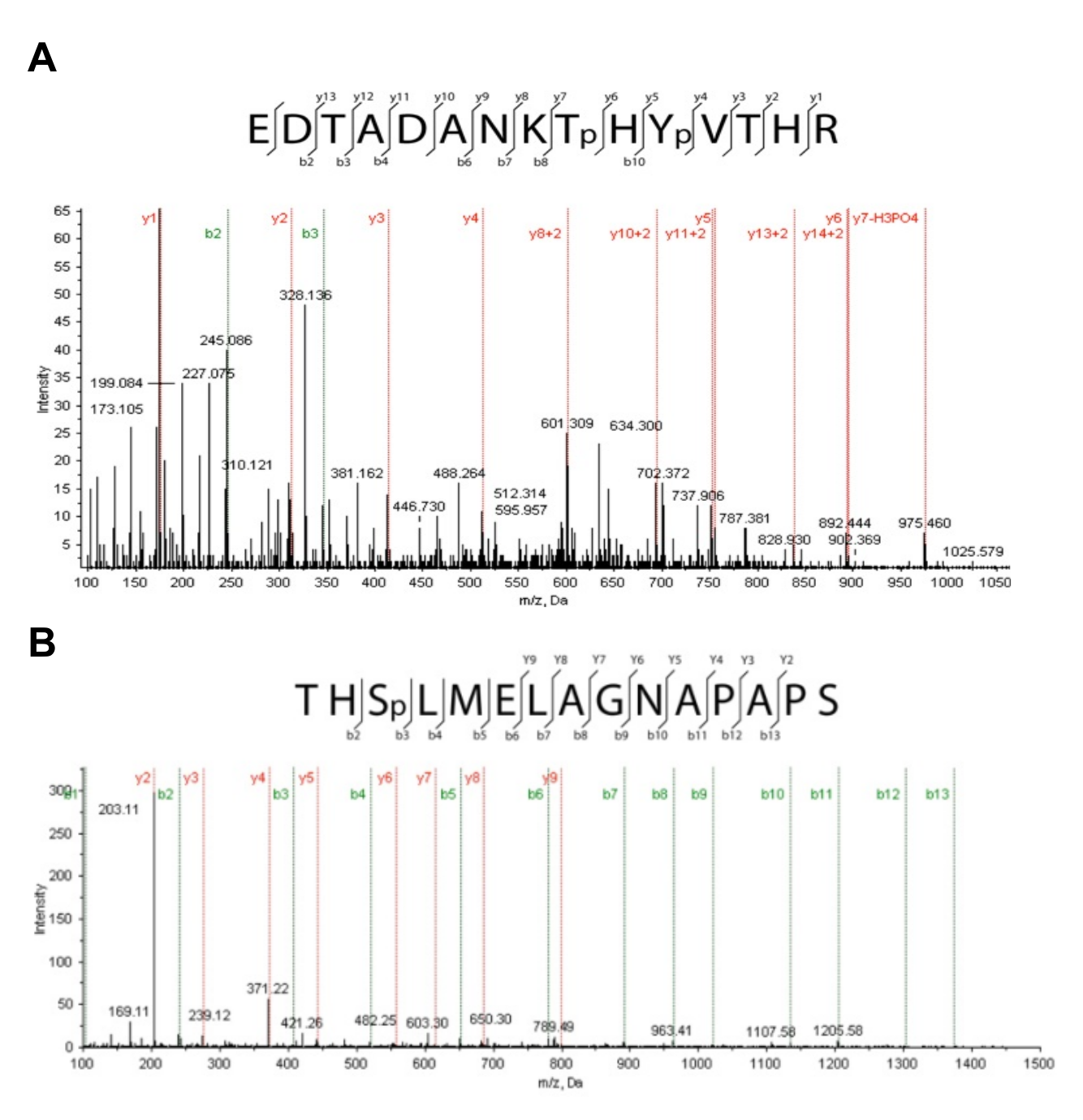

Supplement: Figure S8 — LC-MS/MS analysis of Leishmania total protein extracts from amastigotes. The samples were subjected to phosphoproteomic analysis following titanium dioxide phosphopeptide enrichment and iTRAQ labeling. MS/MS raw data spectra of identified peptides presenting phosphorylation of threonine and tyrosine of the TxY motif (A), and phosphorylation of serine 395 located inside the carboxy terminal extension of MPK10 (B) are shown. (TIF) [file ppat.1004347.s008.tif]
